# Association between white matter alterations and domain-specific cognitive impairment in cerebral small vessel disease: A meta-analysis of diffusion tensor imaging

**DOI:** 10.3389/fnagi.2022.1019088

**Published:** 2022-11-22

**Authors:** Yao Xie, Le Xie, Fuliang Kang, Junlin Jiang, Ting Yao, Guo Mao, Rui Fang, Jianhu Fan, Dahua Wu

**Affiliations:** ^1^Department of Neurology, Hunan Academy of Traditional Chinese Medicine Affiliated Hospital, Changsha, China; ^2^Department of Imaging, Hunan Academy of Traditional Chinese Medicine Affiliated Hospital, Changsha, China; ^3^Office of Academic Research, Hunan Academy of Traditional Chinese Medicine Affiliated Hospital, Changsha, China; ^4^College of Integrated Chinese and Western Medicine, Hunan University of Chinese Medicine, Changsha, China

**Keywords:** small vessel disease (SVD), cognitive impairment, diffusion tensor imaging (DTI), neuroimage, meta-analysis

## Abstract

**Objective:**

To investigate the association between diffusion tensor imaging (DTI) findings and domain-specific cognitive impairment in cerebral small vessel disease (CSVD).

**Methods:**

Databases such as PubMed, Excerpta Medical Database (EMBASE), Web of Science, Cochrane Library, Chinese National Knowledge Infrastructure Databases (CNKI), Wanfang, Chinese Biomedical Literature Database (SinoMed), and Chongqing Chinese Science and Technology Periodical Database (VIP) were comprehensively retrieved for studies that reported correlation coefficients between cognition and DTI values. Random effects models and meta-regression were applied to account for heterogeneity among study results. Subgroup and publication bias analyses were performed using Stata software.

**Results:**

Seventy-seven studies involving 6,558 participants were included in our meta-analysis. The diagnosis classification included CSVD, white matter hyperintensities (WMH), subcortical ischemic vascular disease, cerebral microbleeding, cerebral amyloid angiopathy (CAA), cerebral autosomal dominant arteriopathy with subcortical infarcts and leukoencephalopathy (CADASIL), and Fabry disease. The pooled estimates showed that the fractional anisotropy (FA)-overall exhibited a moderate correlation with general cognition, executive function, attention, construction, and motor performance (*r* = 0.451, 0.339, 0.410, and 0.319), and the mean diffusitivity/apparent diffusion coefficient (MD/ADC)-overall was moderately associated with general cognition, executive function, and memory (*r* = −0.388, −0.332, and −0.303, respectively; *p*_*s*_ < 0.05). Moreover, FA in cingulate gyrus (CG), cerebral peduncle (CP), corona radiata (CR), external capsule (EC), frontal lobe (FL), fornix (FOR), internal capsule (IC), and thalamic radiation (TR) was strongly correlated with general cognition (*r* = 0.591, 0.584, 0.543, 0.662, 0.614, 0.543, 0.597, and 0.571), and a strong correlation was found between MD/ADC and CG (*r* = −0.526), normal-appearing white matter (NAWM; *r* = −0.546), and whole brain white matter (WBWM; *r* = −0.505). FA in fronto-occipital fasciculus (FOF) (*r* = 0.523) and FL (*r* = 0.509) was strongly associated with executive function. Only MD/ADC of the corpus callosum (CC) was strongly associated with memory (*r* = −0.730). Besides, FA in CG (*r* = 0.532), CC (*r* = 0.538), and FL (*r* = 0.732) was strongly related to the attention domain. Finally, we found that the sample size, etiology, magnetic resonance imaging (MRI) magnet strength, study type, and study quality contributed to interstudy heterogeneity.

**Conclusion:**

Lower FA or higher MD/ADC values were related to more severe cognitive impairment. General cognition and executive function domains attracted the greatest interest. The FL was commonly examined and strongly associated with general cognition, executive function, and attention. The CC was strongly associated with memory and attention. The CG was strongly related to general cognition and attention. The CR, IC, and TR were also strongly related to general cognition. Indeed, these results should be validated in high-quality prospective studies with larger sample sizes.

**Systematic review registration:**

http://www.crd.york.ac.uk/PROSPERO, identifier: CRD42021226133.

## Introduction

Cerebral small vessel disease (CSVD) is a chronic, progressive disorder of small arteries, arterioles, capillaries, and venules in the brain resulting from various causes (Peng, 2019). CSVD is the most common cause of vascular cognitive impairment and accounts for 45% of dementia cases (Gorelick et al., 2011). At present, the diagnosis of CSVD mainly depends on computed tomography (CT) or magnetic resonance imaging (MRI). Given that CT has poor sensitivity for detecting brain lesions, MRI is the mainstay of the diagnosis of CSVD. Features observed on MRI include small subcortical infarcts, lacunes, white matter hyperintensities (WMH), perivascular spaces, microbleeds, and brain atrophy (Wardlaw et al., 2013). However, no biomarker is currently available to diagnose cognitive impairment in CSVD.

It is widely acknowledged that CSVD mainly leads to white matter (WM) damage. Growing evidence suggests that WM plays an important role in cognitive decline and dementia (Prins and Scheltens, 2015). Diffusion tensor imaging (DTI) is a newly developed MRI technique used to detect microscopic structural changes in WM and evaluate the integrity of WM fibers by quantifying the asymmetry (fractional anisotropy; FA) and amount of water diffusion (mean diffusivity/apparent diffusion coefficient; MD/ADC). It is widely considered a candidate imaging marker for evaluating cognitive impairment (Pasi et al., 2016). Over the years, many studies have explored cognitive impairment of CSVD by using DTI. However, the relationship between DTI and cognitive disorder in CSVD remains unclear. Indeed, different studies have examined different cognitive domains and brain regions separately. To our knowledge, no meta-analysis has hitherto summarized the association between DTI findings and cognitive outcomes following CSVD. To address this gap, our study explored the association between DTI findings and domain-specific cognitive impairment in CSVD.

## Methods

The meta-analysis was conducted according to the Preferred Reporting Items for Systematic Reviews and Meta-Analyses (PRISMA) guidelines (Moher et al., 2009). This research was registered in PROSPERO (International prospective register of systematic reviews; registration number: CRD42021226133), and the meta-analysis protocol was published online after peer review (Xie et al., 2021).

### Literature search and study eligibility

Databases such as PubMed, Excerpta Medical Database (EMBASE), Web of Science, Cochrane Library, Chinese National Knowledge Infrastructure Databases (CNKI), Wanfang, Chinese Biomedical Literature Database (SinoMed), and Chongqing Chinese Science and Technology Periodical Database (VIP) were retrieved for relevant studies published from 1 January 1994, to 1 August 2021. The detailed search strategy can be found in the published protocol (Xie et al., 2021).

We included studies according to the following criteria: (1) MRI or CT was used to determine CSVD in adults (≥18 years). (2) Participants had mild cognitive impairment or dementia and suffered at least one cognitive domain disorder. (3) FA and/or MD/ADC data were reported for one or more brain regions during DTI analysis. (4) Cognitive testing scores were reported in the studies. (5) Correlations between cognitive testing scores and DTI values were reported (Pearson's *r*, Spearman's rho, or other correlation coefficients). (6) Cohort, case-control, or cross-sectional studies. Exclusion criteria were: (1) Comorbidities such as immune-mediated conditions or other severe diseases (e.g., multiple sclerosis, sarcoidosis, or radiation-induced-encephalopathy). (2) Cognitive disorder was mainly caused by neurodegenerative disorders or other diseases (e.g., Alzheimer's disease, frontotemporal dementia, Lewy body dementia, Parkinson's disease, massive cerebral infarction, trauma, toxic). (3) Reviews, case studies, and studies with very small samples (*N* ≤ 5). (4) DTI data or cognitive testing scores could not be extracted, calculated, or provided by the study authors. (5) Studies that used research data from different articles.

### Study selection and data extraction

Literature search results were imported into NoteExpress software (V3.0). Data extraction was performed independently by three reviewers (YX, LX, and FK). If multiple reports were published from the same study, we included the study with the most up-to-date or comprehensive information. The following data were extracted: publication, general information, study design, participant characteristics, control, comorbidities, type of CSVD, the severity of cognitive impairment, DTI metrics, cognitive testing, the brand of scanner, MRI magnet strength, method of analysis, duration of follow-up, etc. Cognitive outcomes were categorized into eight cognitive domains according to previous studies (Lezak et al., 2012): general cognition, memory, attention, processing speed and working memory, executive function, verbal skills, concept formation and reasoning, construction and motor performance.

### Quality control and bias assessment

The Newcastle–Ottawa Scale (NOS) was used to evaluate the quality of the included cohort or case-control studies (Wells, 2004). It contains eight items, including selection, comparability, exposure, and outcome and the maximum score is 9. The quality of studies was graded as good (≥7), fair (4–6), or poor (<4). The quality of cross-sectional studies was assessed by the Agency for Healthcare Research and Quality (AHRQ) scale (Rostom et al., 2004), which contains 11 items. The studies were assessed as low, moderate, and high quality for scores of 0–3, 4–7, and 8–11, respectively.

### Statistical analysis

Pearson's correlation analysis was applied to assess the relationship between the DTI metrics of each brain region and individual cognitive domain. Spearman's rho was transformed with the equation (*r* = 2sin[r_s_ π/6]) (Rupinski and Dunlap, 1996). Then, summary *r* was calculated with Fisher's *Z*, which was transformed with Pearson's *r* (Borenstein et al., 2009). Effect sizes (summary *r*) of 0.1, 0.3, 0.5, and 0.7 correspond to small, moderate, strong, and very strong effects (Rosenthal, 1996). The beta coefficients were also transformed into Pearson's correlation coefficient (Peterson and Brown, 2005).

The following variables were calculated in meta-regression and subgroup analysis: etiology (arteriosclerosis versus genetic), study type (cohort versus case-control versus cross-sectional studies), magnet strength (1.5T vs. 3.0T), sample size (≤50 vs. >50), and quality of the study (low quality versus moderate quality versus high quality). The random effects model (Der Simonian and Laird method), which is more conservative, was applied in all meta-analyses, since the clinical and methodological conditions differed to some extent in included studies (DerSimonian and Laird, 1986). It has been established that the *Q*-test has poor power to analyze heterogeneity when few studies are included (Higgins and Thompson, 2002). In the present study, heterogeneity among the included studies was assessed by *I*^2^. According to the Cochrane Handbook for Systematic Reviews of Interventions (version 5.1.0), the degree of heterogeneity was defined by the *I*^2^ value. *I*^2^ values of 0%−40% might not be important, and 30–60, 50–90, and 75%−100% represented moderate, substantial, and considerable heterogeneity.

Publication bias was assessed by the funnel plot and Egger's test. A *p*-value of **<** 0.05 was statistically significant. All analyses were performed with Stata version 15.1.

## Results

### Participant and study characteristics

**Figure 1:**
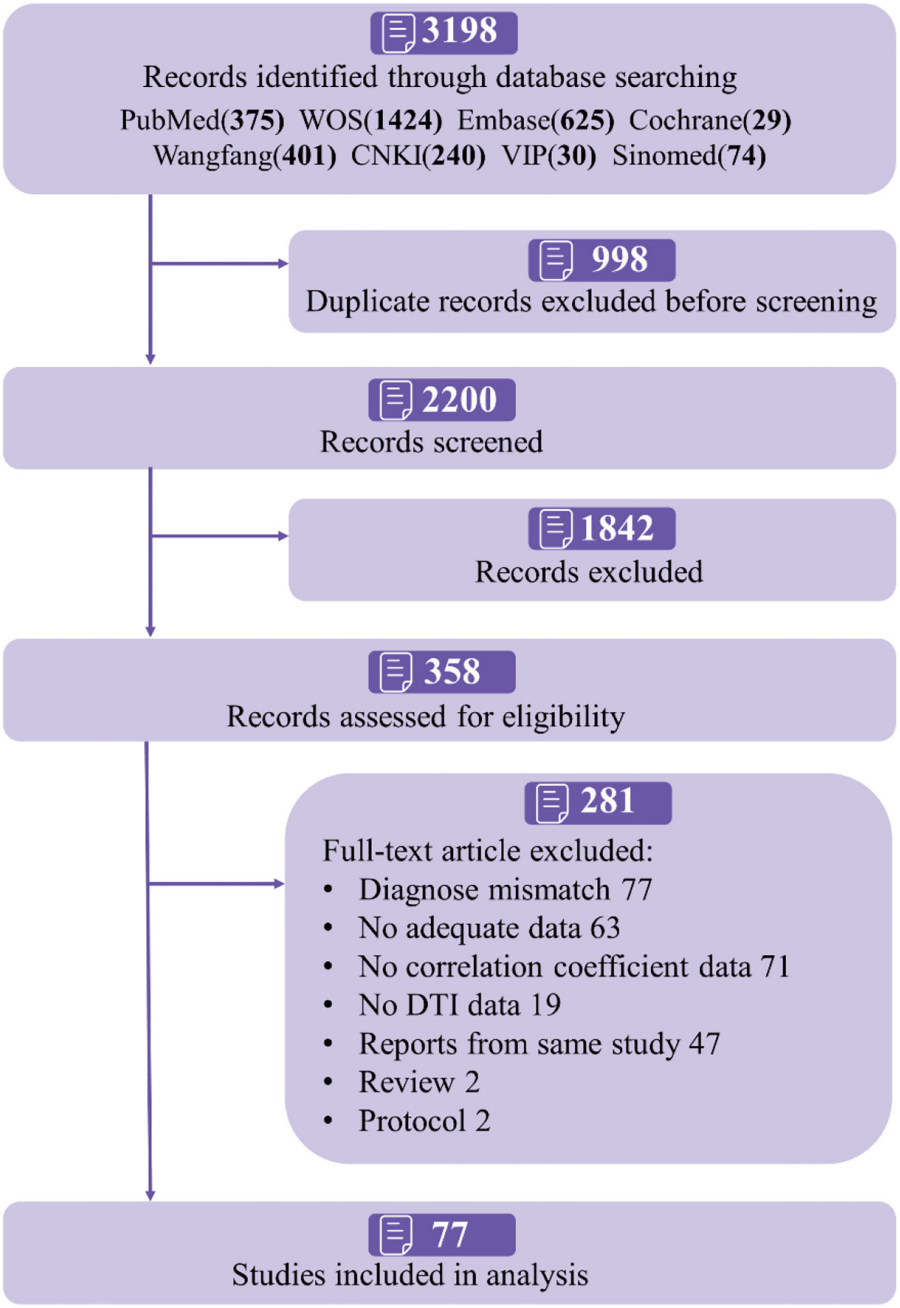
Flowchart of study selection.

Figure 1 shows the selection of studies to be included in the present study. Finally, 77 studies were included for systematic review (Table 1 and Supplementary Table S1 for summary information). The sample size of individual studies ranged from 23 to 801, with a total of 6,558 participants. Among those, 58 studies (75.3%) were conducted in Asia, and the rest were conducted in Europe or America. The included studies enrolled patients with CSVD, WMH, subcortical ischemic vascular disease (SIVD), cerebral microbleeds (CMB), cerebral amyloid angiopathy (CAA), cerebral autosomal dominant arteriopathy with subcortical infarcts and leukoencephalopathy (CADASIL), and Fabry disease. Patients with WMH (36.4%) and SIVD (31.2%) were the most commonly analyzed. In most cases, DTI was performed with 3 Tesla MRI (71.4%), and region of analysis (ROI) analysis was conducted in 52 studies (67.5%). Overall, eight cognitive domains and 38 brain regions were reported, with mild or moderate cognition in most studies (62.3%), while 24 studies did not report participant cognitive status. The average education degree in 37.7% of studies was 10.2 years, and 29 studies did not describe education information.

**Table 1:** Summarized characteristics of the included study.

**Researches**	**Number of studies (%) (*****n*** = **77)**	
**Diagnosis and classification**
CSVD	12 (15.6%)
White matter hyperintensity	28 (36.4%)
Subcortical ischemic vascular disease	24 (31.2%)
Cerebral microbleeds	2 (2.6%)
CADASIL	7 (9.1%)
Cerebral amyloid angiopathy	3 (3.9%)
Fabry	1 (1.3%)
**Study sites**
Asian	58 (75.3%)
Europe and America	19 (24.7%)
**Study type**
Cross-sectional	68 (88.3%)
Cohort	7 (9.1%)
Case–control	2 (2.6%)
**Quality of research**
High quality	6 (7.8%)
Moderate quality	50 (64.9%)
Low quality	21 (27.3%)
**Centers**
Single center	71 (92.2%)
Multiple centers	6 (7.8%)
**Participants**	***N*_studies_(%)**	**Mean or percentage**
Age	75 (97.4%)	64.8 (years)
Male	72 (93.5%)	57%
MMSE	21 (27.3%)	25.8 (scores)
MoCA	32 (41.6%)	23.1 (scores)
Hypertension	37 (48.1%)	53.8%
Smoking	25 (32.5%)	31.3%
Education	46 (59.7%)	10.3 (years)
**MRI**	***N*_studies_(%)**
**DTI metrics**
FA	63 (81.8%)
MD/ADC	59 (76.6%)
*b* = 1000 (DWI)	55 (71.4%)
**Brand of scanner**
General electric	36 (46.8%)
Philips	13 (16.9%)
Siemens	24 (31.2%)
**MRI magnet strength**
1.5 Tesla	20 (26%)
3 Tesla	55 (71.4%)
**Method(s) of analysis**
ROI	52 (67.5%)
Multivariate analysis	20 (26.0%)

### Relationship between FA, MD/ADC, and cognition

#### General cognition

**Figure 2:**
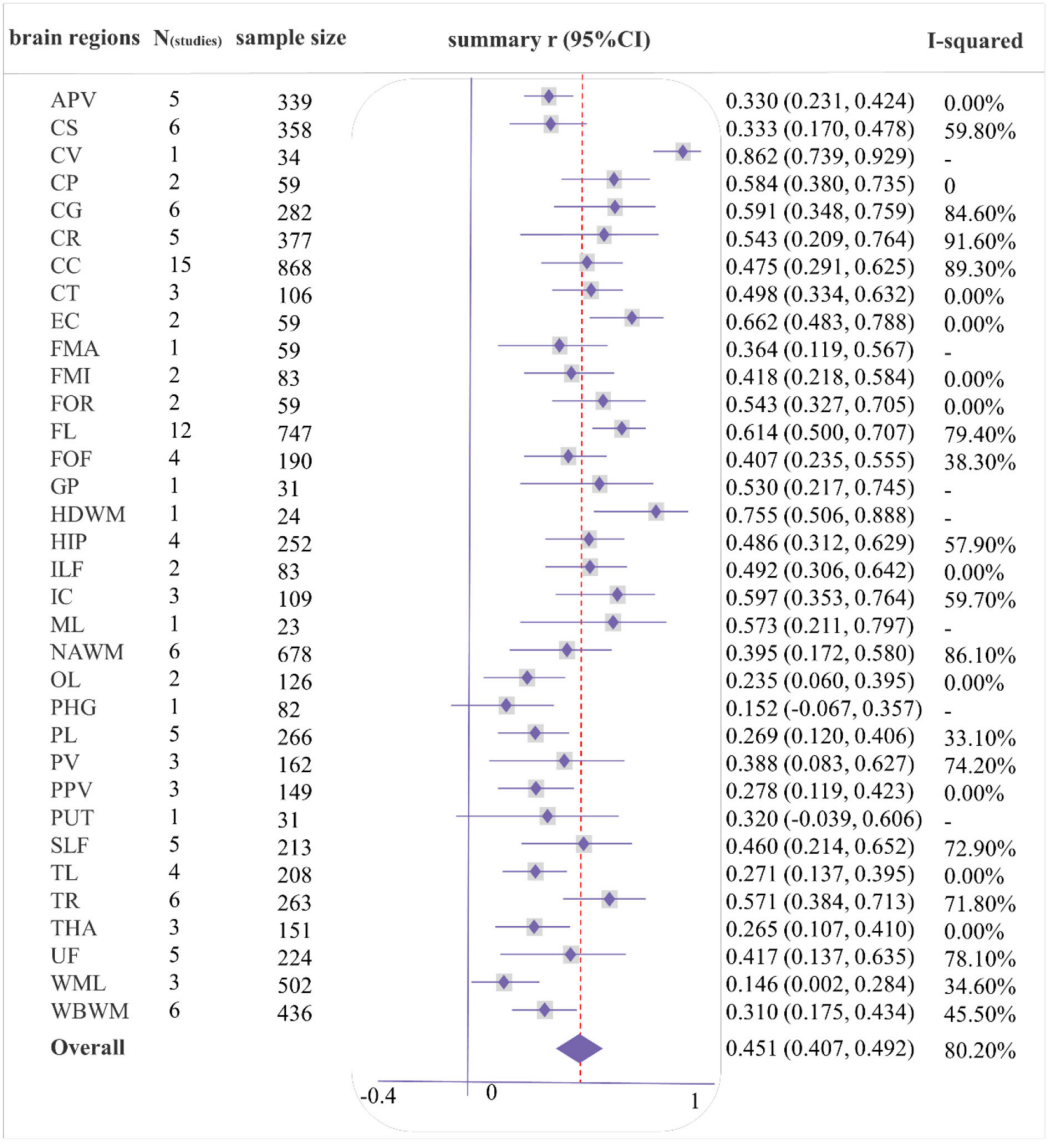
Forest plot of correlations between fractional anisotropy (FA) and general cognition by different brain regions. Summary of abbreviated words in Supplementary Table S5, the following figures are the same as above.

**Figure 3:**
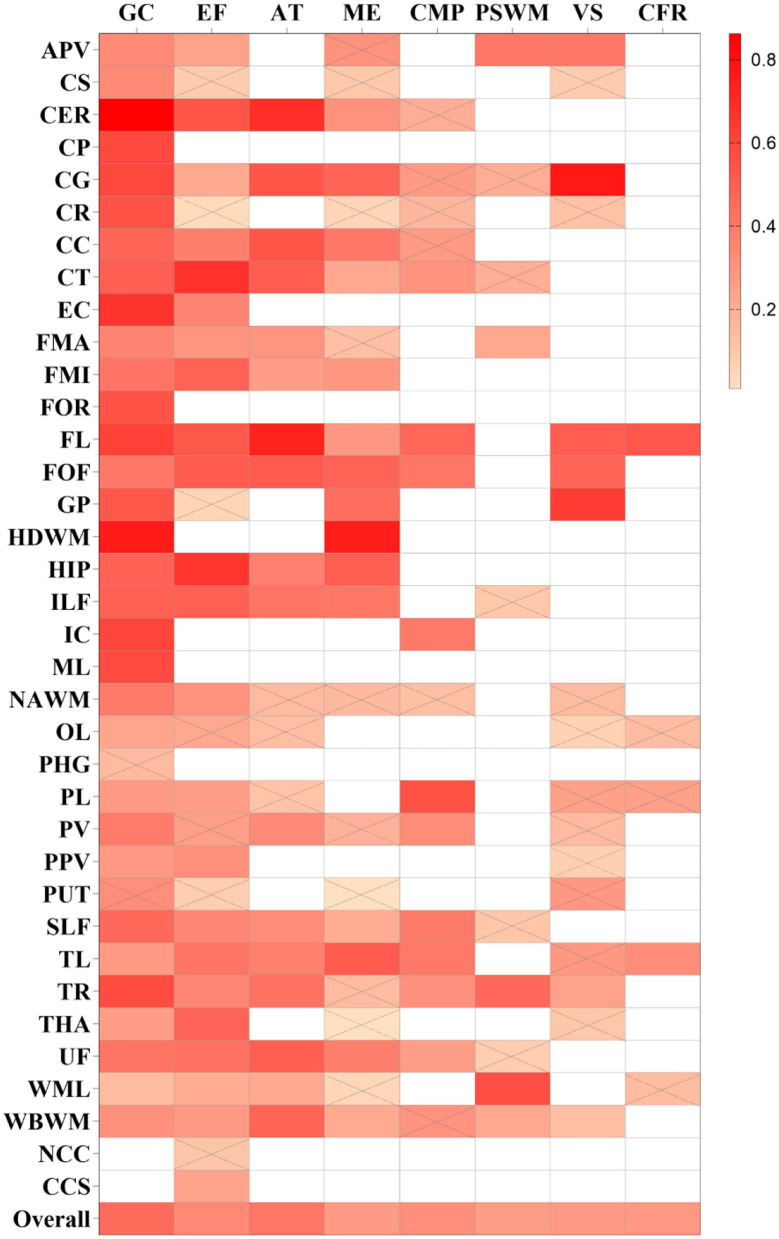
Summary correlation coefficients between different cognitive domains and fractional anisotropy (FA) in varying regions with color scales. Correlation matrix for the *r*-value of meta-analytic tests, the larger *r*-values of squares are with a brighter color. Squares with a white color represent no studies that report the relationship. Squares with gray slashes denote insignificant correlation (*p* > 0.05).

As shown in Figures 2, 3, a moderate correlation was found between FA and general cognition [*r* = 0.451, 95% confidence interval (CI) 0.407 to 0.492, *I*^2^ = 80.2%]. Moreover, a strong correlation was found with cingulate gyrus (CG), corona radiata (CR), frontal lobe (FL), internal capsule (IC), and thalamic radiation (TR) reported in at least three studies, yielding correlation coefficients of 0.591 (0.348, 0.759), 0.543 (0.209, 0.764), 0.614 (0.500, 0.707), 0.597 (0.353, 0.764), and 0.571 (0.384, 0.713), respectively; however, considerable heterogeneity was observed. Other brain regions also showed a strong correlation effect only in one or two studies, such as the cerebellum (CER), cerebral peduncle (CP), external capsule (EC), fornix (FOR), globus pallidus (GP), hemispheric deep white matter (HDWM), inferior longitudinal fasciculus (ILF), and medial lemniscus (ML). These cross-sectional studies were not of high quality, with a relatively small sample size (smaller than 40).

**Figure 4:**
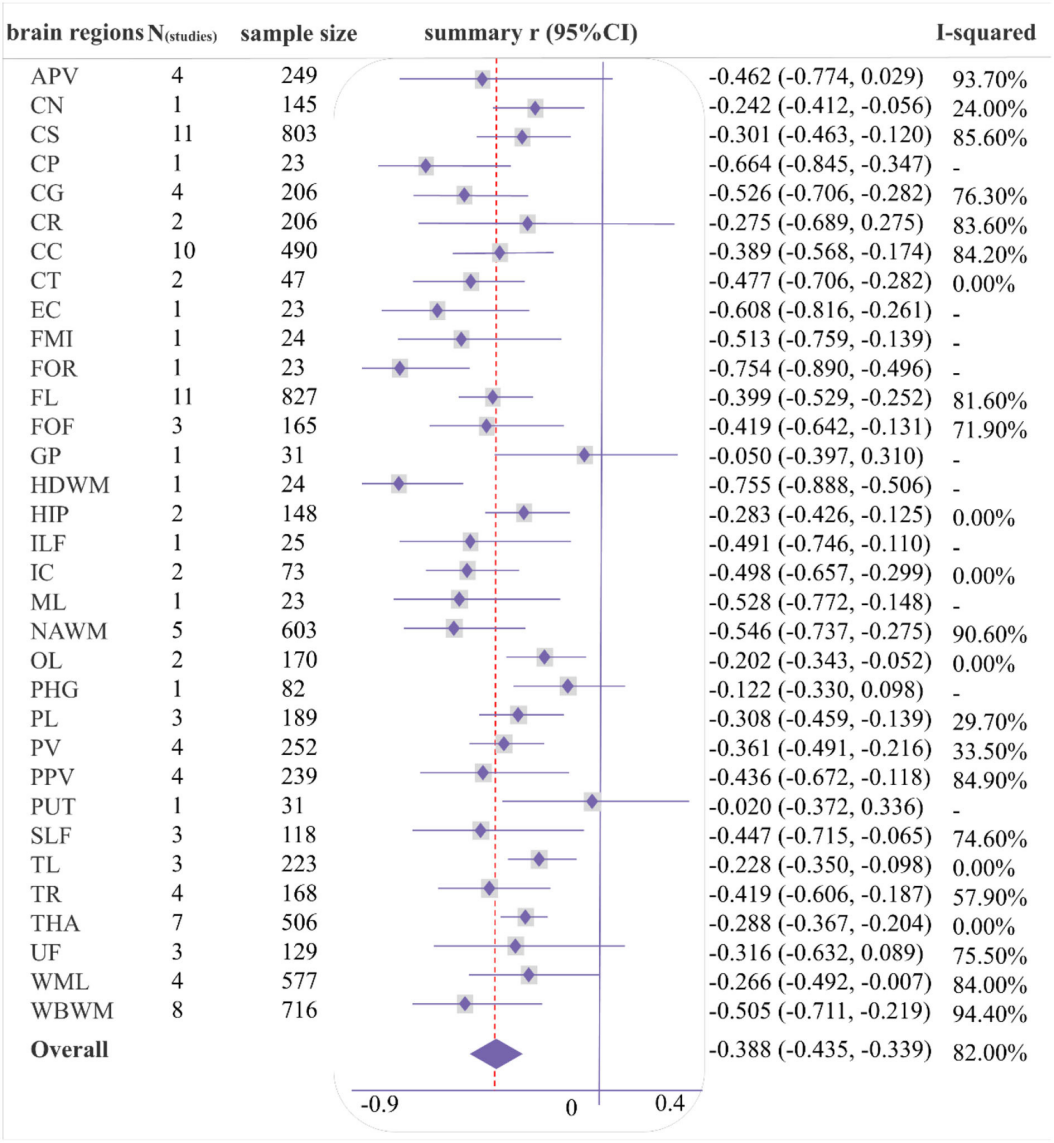
Forest plot of correlations between mean diffusivity/apparent diffusion coefficient (MD/ADC) and general cognition by different brain regions.

**Figure 5:**
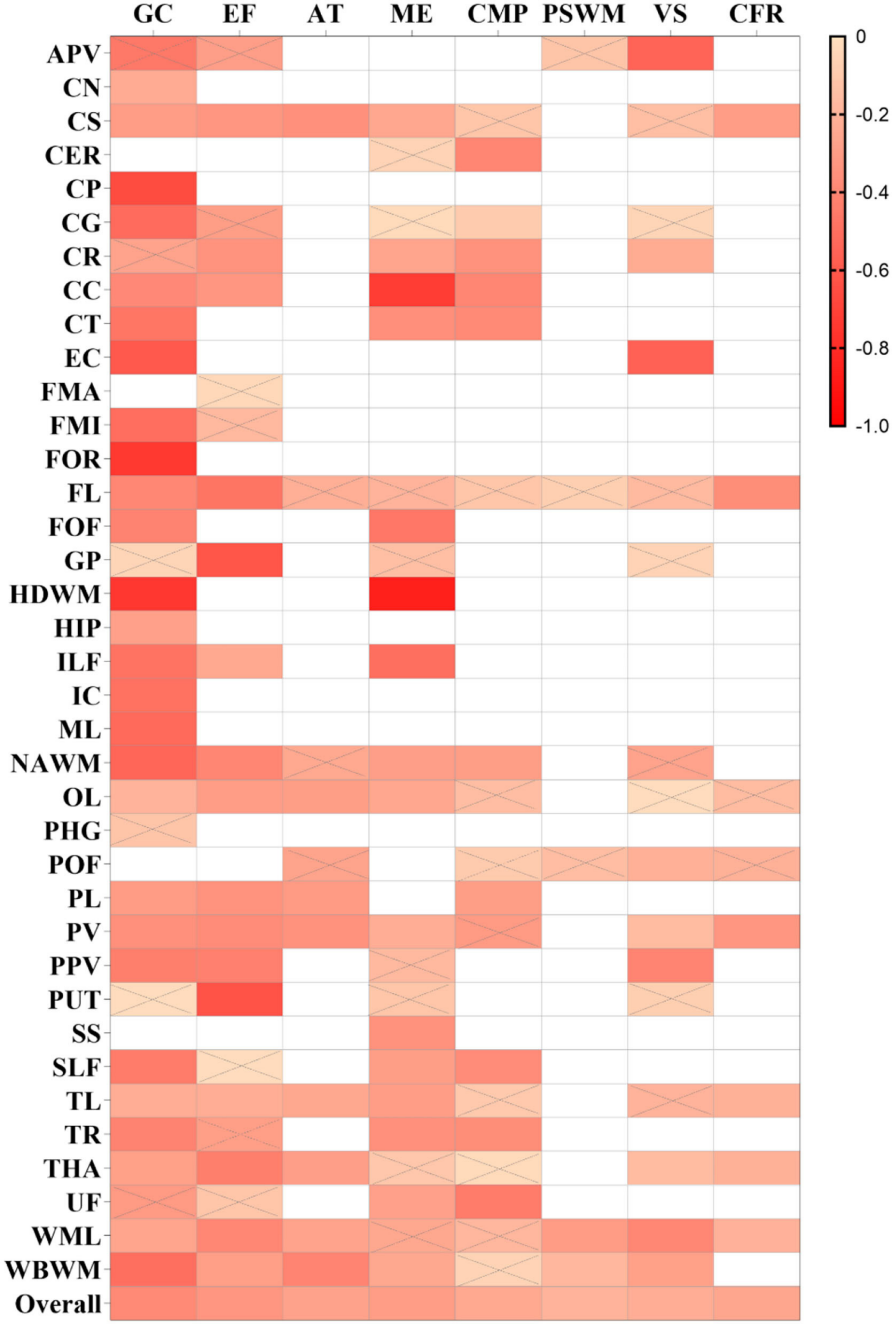
Summary correlation coefficients between different cognitive domains and mean diffusivity/apparent diffusion coefficient (MD/ADC) in varying regions with color scales. The smaller *r* values of squares are displayed in a brighter color. The others are the same as above.

As shown in Figures 4, 5, a moderate correlation was found between MD/ADC and general cognition [*r* = −0.388 (−0.435, −0.339), *I*^2^ = 82%]. Strong correlations were found with CG, normal-appearing white matter (NAWM), and whole brain white matter [WBWM; *r* = −0.526 (−0.706, −0.282); *r* = −0.546 (−0.737, −0.275); *r* = −0.505 (−0.711, −0.219)], which were supported by more than three studies, but marked heterogeneity was observed. Similarly, CP, EC, forceps minor (FMI), FOR, HDWM, and ML presented a strong correlation in low-quality studies and small sample sizes.

#### Executive function

The pooled correlation between FA and executive function was 0.339 (0.296, 0.381), *I*^2^ = 67% (Supplementary Figure S1). FL was reported in five studies and the pooled estimates showed a strong correlation [*r* = 0.523 (0.382, 0.640), *I*^2^ = 40.60%]. Although the FA of CER, corticospinal tract (CT), fronto-occipital fasciculus (FOF), and hippocampus (HIP) was reported in less than three studies, a strong correlation was found with the executive function. Among these, FOF was reported by two studies, with a total sample size of 155, and only one high-quality study was included.

The pooled correlation between MD/ADC and executive function was −0.332 (−0.382, −0.280), *I*^2^ = 71.6% (Supplementary Figure S2). Although GP and putamen (PUT) indicated a strong correlation with executive function in one study, this study was low-quality, including less than 40 participants.

#### Memory

A weak correlation was observed between memory and FA [*r* = 0.276 (0.221, 0.328), *I*^2^ = 64.8%], and a moderate association between MD/ADC and memory [*r* = −0.303 (−0.364, −0.241), *I*^2^ = 64.8%; Supplementary Figures S3, S4]. Several brain regions showed a strong correlation between DTI and memory in one or two studies, including the HDWM, HIP, temporal lobe (TL) for FA, and corpus callosum (CC), HDWM, and ILF for MD/ADC. Among these, the sample size for TL or CC was more than 50, and all studies were of moderate quality. The correlation coefficients for TL and CC were 0.510 (0.289, 0.679) and −0.730 (−0.922, −0.247).

#### Attention

A weak correlation was found between MD/ADC and attention [*r* = −0.278 (−0.357, −0.194), *I*^2^ = 62.9%; Supplementary Figure S5], while no brain regions with a strong correlation between MD/ADC and attention were observed. However, FA exhibited a moderate correlation with attention [*r* = 0.410 (0.327, 0.488), *I*^2^ = 75.8%; Supplementary Figure S6]. FA in CER, CG, CC, CT, FL, and FOF strongly correlated with attention. Two moderate-quality studies with more than 80 participants were available for CG, CC, and FL. The pooled analysis yielded correlation values of 0.532 (0.365, 0.666), 0.538 (0.141, 0.787), and 0.732 (0.608, 0.822).

#### Processing speed and working memory

Fractional anisotropy and MD/ADC yielded a weak correlation in processing speed and working memory [PSWM; *r* = 0.259 (0.176, 0.338), *I*^2^ = 64.4%; −0.202 (−0.310, −0.090), *I*^2^ = 69.6%; Supplementary Figures S7, S8]. Moreover, only FA in WML was strongly correlated with PSWM [*r* = 0.558 (0.331, 0.724)], which was reported by a moderate-quality study involving 50 participants.

#### Verbal skills

FA and MD/ADC also exhibited a weak correlation with verbal skills [*r* = 0.276 (0.177, 0.369), *I*^2^ = 67.6%; *r* = −0.226 (−0.286, −0.164), *I*^2^ = 30%; Supplementary Figures S9, S10]. However, in one study, some brain regions exhibited a strong correlation with verbal skills, including CG, FL, and GP for FA, and anterior periventricular (APV) and EC for MD/ADC. A moderate-quality study reported a correlation coefficient of 0.766 (0.625, 0.859) for CG, while other studies for Fl and GP were low-quality. Compared with APV, the study results for EC were obtained after adjusting for covariates and yielded a correlation coefficient of −0.564 (−0.743, −0.310).

#### Concept formation and reasoning

Overall, the strength of the correlation between FA and concept formation and reasoning (CFR) and between MD and CFR was weak [*r* = 0.277 (0.125, 0.415), *I*^2^ = 36%; *r* = −0.260 (−0.328, −0.188), *I*^2^ = 0%; Supplementary Figures S11, S12). A low-quality study indicated that FA in FL was strongly correlated with CFR [*r* = 0.528 (0.280, 0.709)].

#### Construction and motor performance

The overall strength of correlation between FA and construction and motor performance (CMP) was moderate [*r* = 0.319 (0.243, 0.391), *I*^2^ = 60.3%], while a weak overall correlation was found between MD and CMP [*r* = −0.248 (−0.311, −0.182), *I*^2^ = 49%; Supplementary Figures S13, S14]. The FA in the parietal lobe (PL) was strongly correlated with CMP [*r* = 0.553 (0.327, 0.719)], which was only reported by a moderate-quality study.

### Meta-regression analysis and subgroup analysis

The meta-regression analysis was performed on the whole brain and a few brain regions (*N*_studies_ ≥ 10; Supplementary Table S2) (Schmid et al., 2004). During meta-regression analysis between FA and the whole brain, sample size, etiology, magnet strength, study type, and study quality contributed to the heterogeneity. However, none of these covariates were associated with the heterogeneity of general cognition-related correlations in the CC, FL, and centrum semiovale (CS). Based on the results of the meta-regression analysis, a subgroup analysis was also performed to identify sources of heterogeneity (Supplementary Table S3). We found that the large sample size, 3 Tesla, and genetic etiology accounted for the close relationship between DTI metrics and cognition. However, high-quality prospective studies indicated a weaker correlation.

### Bias and quality assessment

Sixty-eight cross-sectional studies, seven cohort studies, and two case-control studies were included in the meta-analysis (Table 1), including high (*n* = 6), moderate (= 50), and low (*n* = 21) quality. Moreover, 20 studies adjusted their baseline data during statistical analysis. Details of individual studies are provided in Supplementary Table S1. Publication bias was assessed in studies with sufficient sample size and strong correlation. Egger's test detected a significant bias only for analysis of the correlation between the MD (NAMW) and general cognition (*p* = 0.01; Supplementary Figure S15).

## Discussion

In this systematic review and meta-analysis of 77 studies, eight cognitive domains were impacted by CSVD. General cognition (*N*_studies_ = 57) and executive function (*N*_studies_ = 34) were the domains of greatest interest in selected studies (Supplementary Figure S16). It is well established that executive function is the main damaged cognitive domain of CSVD (Peng, 2019; Litak et al., 2020). In the present study, FA and MD/ADC were significantly associated with these eight cognitive domains (all *p* < 0.05). During DTI analysis, low fractional anisotropy and high mean diffusivity generally reflect lower microstructural connectivity. Our meta-analysis showed that lower FA or higher MD values were related to more severe cognitive impairment.

According to the Vascular Behavioral and Cognitive Disorders (VASCOG) statement, it is recommended to evaluate multiple cognitive domains for CSVD (Sachdev et al., 2014). Different cognitive domains exhibited different correlations with DTI metrics in our study. For instance, FA-overall exhibited a moderate correlation with general cognition, executive function, attention, construction, and motor performance (*r* = 0.451, 0.339, 0.410, and 0.319, respectively). Moreover, MD/ADC-overall exhibited a moderate correlation with general cognition, executive function, and memory (*r* = −0.388, −0.332, and −0.303) and a weak correlation with the remaining domains. This finding provided the rationale for applying DTI to evaluate cognition in CSVD.

According to the Journal of the American College of Cardiology (JACC) scientific expert panel, it is very difficult to improve cognitive disorder during the late stages (Iadecola et al., 2019), emphasizing the importance of early diagnosis and treatment of cognitive impairment. Over the years, significant emphasis has been placed on biological markers for early identification and intervention in white matter damage. DTI can detect early tissue alterations, even in white matter appearing normal on conventional imaging (van den Brink et al., 2022). Some cognitive domains are affected during early cognitive impairment of CSVD, including executive function and attention (van der Flier et al., 2018; Peng, 2019). In this meta-analysis, executive function and attention were closely associated with DTI metrics.

Progressive cognitive impairment, gait disorders, and depression are the main clinical features of CSVD. Accordingly, emphasis should be placed on the specific microscopic structure changes associated with different impaired cognitive domains. Among the 77 included studies, 37 brain regions were explored. We found that white matter damage in specific areas was strongly associated with several cognitive domains (Supplementary Table S4). For instance, FL was strongly associated with the executive function and attention domains (*r* = 0.523, 0.732), and CC was strongly associated with the memory domain (*r* = −0.730). The mechanism underlying frontal lobe (FL) damage may involve dysregulation in the frontal–subcortical loop. Damage to the frontal–subcortical network can occur in the form of white matter lesions or microbleeds affecting pathways that connect cortical and subcortical regions, which result in cognitive impairments in executive function and attention (Ye and Bai, 2018). It is widely thought that white matter tracts in the CC harbor commissural fibers connecting the frontal lobe (FL) and other cortical areas, which can be damaged at the early stage of CSVD (Qiu et al., 2021). Overwhelming evidence indicates that brain network disorders lead to cognitive impairment of CSVD, and damaged network structure accounts for the association between CSVD neuroimaging lesions and cognitive deficits (Vasquez and Zakzanis, 2015). DTI technology may provide new opportunities to deepen our understanding of disconnection syndromes. Moreover, connectomics developed by DTI can be used to more specifically analyze the regions and functional connections in the cognitive impairment of CSVD.

Significant heterogeneity in participant characteristics and methodology was observed in the included studies. Random effects models and meta-regression models were applied to decrease heterogeneity. The higher quality and prospective design of studies led to more conservative and relatively lower correlation results, which yielded more reasonable conclusions. However, a larger sample size led to a more significant correlation. Current evidence suggests that genetic causes, like CADASIL, may damage white matter more severely, suggesting that DTI findings in hereditary CSVD exhibit a more obvious relationship with cognitive impairment (Cannistraro et al., 2019). In selected studies, differences in parameters, method of analysis, the brand of scanner, and MRI magnet strength led to significant interstudy heterogeneity. In this respect, 3.0 Tesla provided a larger effect size than 1.5 Tesla during meta-regression analysis which may be attributed to the better accuracy of 3.0 Tesla (Soares et al., 2013).

A comprehensive search and methodological effort were performed to summarize reliable correlation coefficients and all related brain regions. The protocol of meta-analysis had been published by peer review. In addition, different patient populations were investigated in our meta-analysis, including CSVD, WMH, SIVD, CMB, CAA, CADASIL, and Fabry disease. However, there were several limitations. First, minimizing heterogeneity attributed to clinical or methodological sources of bias was challenging, and subgroup analyses could not be performed for age, education level, and disease severity. Besides, most included studies were cross-sectional, mostly low-quality, and based on small sample sizes, which decreased the strength of our findings. Moreover, findings for some brain regions were roughly summarized and not comprehensively analyzed. For example, the splenium, body, and genu of CC were all described as CC. Moreover, the clinical assessment scales applied for cognitive tests varied in different studies.

Our findings suggest that DTI technology exhibits significant clinical value for the assessment of white matter alternations due to CSVD. High-quality prospective studies based on large sample sizes are warranted to explore the key regions and brain networks of cognitive disorders using DTI and to establish clinical value for domain-specific cognitive impairment.

## Conclusion

In conclusion, the damaged regional white matter detected by DTI is associated with domain-specific cognitive deficits in CSVD. Lower FA or higher MD values are related to more severe cognitive impairment. General cognition and executive function domains are of greatest interest in the current literature. The frontal lobe (FL) was strongly associated with general cognition, executive function, and attention. The corpus callosum (CC) was strongly associated with memory and attention. The cingulate gyrus (CG) was strongly related to general cognition and attention. The CR, IC, and TR were also strongly related to general cognition. However, sample size, etiology, MRI magnet strength, study type, and study quality contributed to heterogeneity in our study. Accordingly, our results should be further validated in prospective studies with high-quality and larger sample sizes. Future research should aim to make uniform the standard specification, analysis method, and reference value of DTI to increase the diagnostic value of DTI for cognitive impairment in CSVD.

## Data availability statement

The original contributions presented in the study are included in the article/Supplementary material, further inquiries can be directed to the corresponding author.

## Author contributions

The concept of this research was proposed by YX. The literature search, article screen, data extraction, and analysis were contributed by YX, LX, and FK. The protocol was drafted by YX, JJ, TY, RF, GM, and JF. The intellectual content was revised by YX and DW. All authors approved the final version of the manuscript.

## Funding

This research was supported by the General project of the Hunan Provincial Health Commission (No. 202218014505), the General project of Hunan Administration of Traditional Chinese Medicine (D2022060), the Open Fund for First-class Disciplines of Integrated Traditional Chinese and Western Medicine (No. 2020ZXYJH10), the Hunan Academy of Traditional Chinese Medicine Foundation (No. 201920), the Foundation of China Center for Evidence-Based Traditional Chinese Medicine (No. 2019XZZX-NB004), the National Key Research and Development Project (No. 2018YFC1704904), and Natural Science Foundation of Hunan Province (No. 2022JJ40241).

## Conflict of interest

The authors declare that the research was conducted in the absence of any commercial or financial relationships that could be construed as a potential conflict of interest.

## Publisher's note

All claims expressed in this article are solely those of the authors and do not necessarily represent those of their affiliated organizations, or those of the publisher, the editors and the reviewers. Any product that may be evaluated in this article, or claim that may be made by its manufacturer, is not guaranteed or endorsed by the publisher.
