# Supplementary material for: Association between white matter alterations and domain-specific cognitive impairment in cerebral small vessel disease: A meta-analysis of diffusion tensor imaging
Source: Front Aging Neurosci. 2022 Nov 22;14:1019088. doi: 10.3389/fnagi.2022.1019088 (PMC9722766; doi:10.3389/fnagi.2022.1019088)
Supplement: Supplementary file 1 [file Table_1.docx]

**Supplementary table 1: Trail characteristics**

**Category I: White matter hyperintensity (N=28)**

| **Research ID** | **Country** | **Sample size** | **Study type** | **Quality** | **Severity** | **Male**  **(%)** | **Age** | **Education** | **Comorbidity** | **Cognition** | **Brand (MRI)** | **Magnet strength** | **Analysis** | **Statistics** |
| --- | --- | --- | --- | --- | --- | --- | --- | --- | --- | --- | --- | --- | --- | --- |
| O’Sullivan et al.(2004)^[1]^ | UK | 55 | Cross-sectional | Moderate | N | 65% | 70.2±8.4 | 10.8±3.6 | Hypertension:65%, Diabetes:8% | N | GE | 1.5 T | ROI | Pearson |
| Zhang et al.(2006)^[2]^ | China | 77 | Cross-sectional | Low | Mantyla:  1-3 | 73% | 67.5 | N | N | N | GE | 1.5 T | ROI | Pearson |
| Nave et al.(2007)^[3]^ | Italy | 36 | Cross-sectional | Moderate | Fazekas:  4±1.3 | 42% | 77±4.5 | N | N | MMSE:  26±3.9 | Philips | 1.5 T | VBM | Spearman |
| Baptista et al.(2008)^[4]^ | Portugal | 30 | Cross-sectional | Moderate | N | 59% | 72.6±5.2 | N | N | N | GE | 1.5 T | ROI | Spearman |
| Nitkunan et al.(2008)^[5]^ | UK | 35 | Cross-sectional | Moderate | modified Fazekas:  2-3 | 69% | 68.8±9.3 | N | Hypertension:97%, Diabetes:29%,  Hyperlipidaemia:66% | N | GE | 1.5 T | Histogram | Pearson |
| Luan et al.(2009)^[6]^ | China | 49 | Cross-sectional | Low | Wahlund:1-3 | 61% | 65.1±7.2 | N | N | MMSE: 24.1±5.4 | GE | 3.0 T | ROI | Pearson |
| Qu et al.(2010)^[7]^ | China | 90 | Cross-sectional | Moderate | Mantylita:1-3 | 68% | 67.5 | N | N | N | GE | 3.0 T | ROI | Pearson |
| Yuan et al.(2010)^[8]^ | China | 32 | Cross-sectional | Low | Mantylita:1-3 | 47% | 62.75 | N | N | N | GE | 1.5 T | ROI | Pearson |
| Li et al.(2012)^[9]^ | China | 40 | Cross-sectional | Moderate | N | 58% | 65.5±7.4 | 7.3 ± 5.4 | Smoking:55% | N | GE | 1.5 T | ROI | Spearman |
| Otsuka et al.(2012)^[10]^ | Japan. | 24 | Cross-sectional | Moderate | Fazekas:3 | 58% | 74±6 | N | Hypertension:100%, Diabetes: 16% | MMSE: 25.1±6.0 | Siemens | 3.0 T | ROI | Spearman |
| Zhang et al.(2013)^[11]^ | China | 31 | Cross-sectional | Low | N | 58% | 67.6±7.2 | ≥9 | N | N | Siemens | 3.0 T | ROI | Pearson |
| Sun et al.(2014)^[12]^ | China | 100 | Cross-sectional | Moderate | ARWMC:1-4 | 39% | 64.1±7.0 | 10.4±3.3 | Hypertension:22.2%, Diabetes:15%,  Hyperlipidaemia:34.1%,Smoking:11% | MMSE: 26.7±2.3 | Siemens | 3.0 T | TBSS | Pearson |
| Pang et al.(2014)^[13]^ | China | 66 | Cross-sectional | Low | N | N | ＞50 | N | N | N | Philips | 1.5 T | ROI | Spearman |
| Ding et al.(2015)^[14]^ | China | 34 | Cross-sectional | Moderate | N | 50% | 63.2±4.8 | 8.55±2.4 | Hypertension:22.7%, Diabetes:33.6%, Hyperlipidaemia:3% | MOCA: 24.5±3.8 | Philips | 3.0 T | ROI | Pearson |
| Pasi et al.(2015)^[15]^ | Italy | 76 | Cross-sectional | High | Fazekas:  2-3 | 55% | 75.1±6.8 | 8.0±4.3 | N | MoCA: 18.9±5.7 | Philips | 1.5 T | ROI | Spearman |
| He et al.(2016)^[16]^ | China | 50 | Cross-sectional | Low | Fazekas:  2-3 | 58% | 65±8.5 | 11.5±3.3 | Hypertension:41.7%, Diabetes:26.7%, Smoking:17.5% | MoCA: 25.5±3.5 | Siemens | 3.0 T | ROI | Pearson |
| Yuan et al.(2017)^[17]^ | China | 100 | Cross-sectional | Moderate | N | 48% | 70.5±5.2 | 8.3±3.9 | Hypertension:63.5%, Diabetes:23%, Smoking:20% | N | Siemens | 3.0 T | ROI | Pearson |
| Li et al.(2017)^[18]^ | China | 59 | Cross-sectional | Low | Fazekas:  1-3 | N | N | N | N | MOCA: 22.8 ±4.7 | Siemens | 3.0 T | ROI | Pearson |
| He et al.(2017)^[19]^ | China | 58 | Cross-sectional | Moderate | N | 62% | 68.7±18.3 | N | N | MOCA: 23.7±5.0 | Siemens | 3.0 T | ROI | Pearson |
| Zhang et al.(2019)^[20]^ | China | 90 | Cross-sectional | Moderate | N | 54% | 65.7 ± 15 | 11.3 ± 3.8 | Hypertension:30%, Diabetes:25%, Hyperlipidaemia:23.3%, Smoking:32.5% | MoCA: 20.5±4.8 | GE | 3.0 T | ROI | Pearson |
| Wang L et al.(2020) ^[21]^ | China | 80 | Cross-sectional | Low | N | 55% | 66. 5±5.9 | 9. 5±2.5 | Hypertension:18.8%, Diabetes:17.5% | MoCA: 22.2±3.6 | GE | 1.5 T | ROI | Pearson |
| Soriano et al.(2014) ^[22]^ | Spain | 96 | Cross-sectional | High | WM volume:  544.1±56 | 41% | 59.7±3.3 | 8±2.8 | Hypertension:55.3%, Diabetes:15.7%, Hyperlipidaemia:60.6%, Smoking:14.4% | MMSE: 29.2±1.5 | Siemens | 3.0 T | TBSS | multiple linear regression |
| Zhong et al.(2017) ^[23]^ | China | 75 | Cross-sectional | Moderate | WML volume:  30.7±24.4 | 48% | 67.05±9.62 | 6.44± 4.75 | Hypertension:69.3%, Diabetes:22.7%, Hyperlipidaemia:25.3%,Smoking:21.3% | MMSE: 25.9±3.5 | GE | 3.0 T | ROI | multiple linear regression |
| Wei et al.(2019) ^[24]^ | China | 161 | Cross-sectional | Moderate | N | 52% | 60.9 ±8.5 | 11.8±3.1 | Hypertension:49.5%, Diabetes:19.1%, Hyperlipidaemia:19.9%,Smoking:33% | MOCA: 22.6±4 | Siemens | 3.0 T | TBSS | multivariate regression analysis |
| Wang S et al.(2020) ^[25]^ | China | 60 | Cross-sectional | Moderate | WML volume:  21.5±16.4 | 50% | 64.8±9.6 | N | Hypertension:61.7%, Diabetes:15%, Hyperlipidaemia:3.3%, Smoking:26.7% | MOCA: 23.5±3.4 | GE | 3.0 T | TBSS | multiple regression models |
| Xing et al.(2021) ^[26]^ | China | 77 | Cross-sectional | Moderate | Fazekas:  2-3 | 48% | 66.4±6.9 | 11.1±2.66 | Hypertension:74%, Diabetes:24.7%, Hyperlipidaemia:54.5% | MMSE: 26.9±2.37 | Siemens | 3.0 T | ROI | multivariate linear regression |
| Wang Z et al.(2020) ^[27]^ | China | 94 | Cross-sectional | Moderate | N | 58% | 59.6±11 | 12.3±3.3 | N | CDR: 0.46±0.26 | N | N | TBSS | Spearman |
| Chen et al.(2020) ^[28]^ | China | 70 | Cross-sectional | Moderate | Fazekas:  2-3 | 55% | 64.7± 2.5 | 11.8±1 | N | MOCA: 23.8±2.5 | Philips | 3.0 T | ROI | partial correlation |

- “N” represents data not reported, the following are the same.

**Category II: Subcortical ischemic vascular disease (N=24)**

| **Research ID** | **Country** | **Sample size** | **Study type** | **Quality** | **Severity** | **Male**  **(%)** | **Age** | **Education** | **Comorbidity** | **Cognition** | **Brand (MRI)** | **Magnet strength** | **Analysis** | **Statistics** |
| --- | --- | --- | --- | --- | --- | --- | --- | --- | --- | --- | --- | --- | --- | --- |
| Zhou et al.(2008) ^[29]^ | China | 38 | Cross-sectional | Moderate | N | N | 70.4±7.9 | 6.2±3.1 | N | MMSE: 20.3±4.9 | Philips | 3.0 T | Histograms | Spearman |
| Yuan et al.(2009) ^[30]^ | China | 60 | Cross-sectional | Low | N | 45% | 67.9±10.3 | N | N | MMSE: 20.2±1.6 | Philips | 3.0 T | ROI | Pearson |
| Xu et al.(2010) ^[31]^ | China | 42 | Cross-sectional | Moderate | N | 81% | 69.2±7.8 | 10.7±3.3 | Hypertension:78.6%, Diabetes:26.2%, Hyperlipidaemia:28.6%,Smoking:14.3% | MMSE: 27.3±3.2 | Philips | 3.0 T | ROI | Pearson |
| Zhang et al.(2011) ^[32]^ | China | 52 | Cross-sectional | Moderate | N | 48% | 65.1±8.8 | 8.4±1.4 | Hypertension:78%, Diabetes:28%, | N | GE | 3.0 T | ROI | Pearson |
| Dong et al.(2011) ^[33]^ | China | 51 | Cross-sectional | Moderate | Wahlund:7.9±5.4 | 73% | 71.1±9.3 | 7.5±2.3 | N | N | GE | 3.0 T | ROI | Pearson |
| Lin et al.(2015) ^[34]^ | China | 50 | Cross-sectional | Moderate | N | 60% | 72±7.4 | 10.75 | N | MoCA: 23.1±4.3 | GE | 3.0 T | ROI | Spearman |
| Pan et al.(2015) ^[35]^ | China | 105 | Cross-sectional | Low | ARWMC:1-3 | 63% | 68.6±6.8 | N | N | MoCA: 22.1±4.1 | GE | 3.0 T | ROI | Pearson |
| Chen et al.(2018) ^[36]^ | China | 24 | Cross-sectional | Moderate | N | 63% | 66.8± 8.2 | 9.8±3.5 | N | MoCA: 22.4±6.1 | GE | 3.0 T | TBSS | Spearman |
| Liu XS et al.(2019) ^[37]^ | China | 73 | Cross-sectional | Moderate | WML volume: 7.16± 1.8 | 60% | 69.6±5.5 | 10.1±2.5 | N | MMSE: 26.±1.1 | GE | 3.0 T | TBSS | Spearman |
| Liao et al.(2019) ^[38]^ | China | 48 | Cross-sectional | Moderate | WML volume: 69.4±37.6 | 58% | 68.8±7.7 | 9.8±3.3 | Hypertension:85.4%, Diabetes:43.8%, Hyperlipidaemia:22.9% | MoCA: 17.9±4.3 | GE | 3.0 T | ROI | Pearson |
| Qiao et al.(2021) ^[39]^ | China | 41 | case–control | Moderate | N | 41% | 63.4±2.5 | 12.5±0.8 | Hypertension:30.4%, Diabetes:31.4%, Hyperlipidaemia:35.6%,Smoking:31.4% | MOCA:27.2±1 | Siemens | 3.0 T | ROI | Pearson |
| Guo et al.(2017) ^[40]^ | China | 46 | Cross-sectional | Low | N | 46% | 68.7±5.5 | 8.9±2.6 | Hypertension:,Diabetes:,Hyperlipidaemia:,Smoking: | MMSE: ≥24 | Siemens | 3.0 T | ROI | Spearman |
| D'Souza et al.(2018) ^[41]^ | India | 60 | Cross-sectional | Moderate | Fazekas  ≥2 | 53% | 66.3±6.5 | N | N | N | Siemens | 3.0 T | DTT | Pearson |
| Zhao et al.(2018) ^[42]^ | China | 84 | Cross-sectional | Moderate | N | 71% | 66.4±4.6 | 10.7±3.1 | Hypertension:71.2%, Diabetes:22%, Hyperlipidaemia:9.2%, Smoking:38.4% | N | GE | 3.0 T | ROI | Pearson |
| Li et al.(2020) ^[43]^ | China | 80 | Cross-sectional | Low | N | 51% | 57.3±2.8 | N | Hypertension:22.5%, Diabetes:17.5%, Hyperlipidaemia:32.6% | N | Siemens | 1.5 T | ROI | Pearson |
| Brandhofe et al.(2021) ^[44]^ | Germany | 57 | Cross-sectional | Moderate | WML volume: 20±17.8 | 47% | 70.6±9.7 | N | N | Global cognitive index: –0.4±0.8 | Siemens | 3.0 T | ROI | Pearson |
| Lawrence et al.(2014) ^[45]^ | UK | 160 | Cross-sectional | High | Fazekas: 2.4±2.5 | 59% | 70.2±9.6 | N | Hypertension:70.9%, Diabetes:9.9%, Smoking:50.8% | N | GE | 1.5 T | Histogram | multiple linear regression |
| Uden et al.(2015) ^[46]^ | Netherlands | 398 | cohort | High | WML volume: 6.0±3 | 57% | 64.5±8.5 | primary: 8.3% | N | MMSE: 28.3±1.6 | Siemens | 1.5 T | ROI | regression model |
| Wu et al.(2016) ^[47]^ | China | 44 | cohort | Moderate | Lacune Count: 2.63±1.52 | 57% | 61.8±8.4 | 10.7±3.6 | Hypertension:64.8%, Diabetes:30.7%, Smoking:41.5% | MoCA: 24.6+2.8 | GE | 3.0 T | ROI | Linear regression analysis |
| Qi et al.(2021) ^[48]^ | China | 53 | Cross-sectional | Moderate | Fazekas: ≥ 2 | 72% | 61.2±6.7 | 11±2.8 | N | MoCA: 24±3.8 | Siemens | 3.0 T | ROI | Partial correlation analyses |
| Tu et al.(2017) ^[49]^ | China/Taiwan | 68 | Cross-sectional | Moderate | N | 63% | 71.7±11.8 | 7±4.6 | Hypertension:24.3%, Diabetes:17.2% | MMSE: 20.6±5.6 | GE | 3.0 T | ROI | Partial correlation |
| Huang et al.(2021) ^[50]^ | China | 244 | Cross-sectional | Moderate | Fazekas: 2-3 | 52% | 65.6±8.5 | 11.3± 3.7 | Hypertension:57.6%, Diabetes:24.3%, Hyperlipidaemia:21.3%,Smoking:23.1% | Z-MoCA: 0.3±1 | Philips | 3.0 T | ROI | Multiple linear regression |
| Qiu et al.(2021) ^[51]^ | China | 183 | Cross-sectional | Moderate | Normal WML: 0.95±0.88 | 79% | 65.3±7.7 | 10.7±3 | N | MoCA: 23.7±3.5 | GE | 3.0 T | ROI | Partial correlations |
| Du et al.(2021) ^[52]^ | China | 202 | Cross-sectional | Moderate | N | 74% | 64.7±7.3 | 10.7±3 | Hypertension:75.6%, Diabetes:37.8%, Hyperlipidaemia:9.6%, Smoking:53.6% | MoCA: 24.5±3.2 | GE | 3.0 T | TBSS | partial correlation |

**Category III: CSVD (N=12)**

| **Research ID** | **Country** | **Sample size** | **Study type** | **Quality** | **Severity** | **Male**  **(%)** | **Age** | **Education** | **Comorbidity** | **Cognition** | **Brand (MRI)** | **Magnet strength** | **Analysis** | **Statistics** |
| --- | --- | --- | --- | --- | --- | --- | --- | --- | --- | --- | --- | --- | --- | --- |
| Chen et al.(2014) ^[53]^ | China | 30 | Cross-sectional | Low | N | 67% | 65.5±8.1 | N | N | MOCA: 22.8±4.5 | GE | 1.5 T | ROI | Spearman |
| Dai et al.(2015) ^[54]^ | China | 51 | Cross-sectional | Low | N | 59% | 62.7±12 | 10.5±4.6 | N | MoCA: ＜26 | Philips | 3.0 T | ROI | Spearman |
| Liu JP et al.(2016) ^[55]^ | China | 46 | Cross-sectional | Low | N | N | N | N | N | MoCA: ＜26 | GE | 3.0 T | ROI | Pearson |
| Hu et al.(2017) ^[56]^ | China | 65 | Cross-sectional | Low | N | 55% | 64.9±6.7 | N | N | MoCA: 24.3±6.5 | GE | 3.0 T | ROI | Pearson |
| Liu DT et al.(2019) ^[57]^ | China | 82 | Cross-sectional | Moderate | N | 57% | 64±8.2 | 8.6±2.3 | Hypertension:65.3%, Diabetes:38.3%, Hyperlipidaemia:50.2%,Smoking:38.4% | MoCA: 24.5±3.7 | Siemens | 3.0 T | DKI | Pearson |
| Zhao et al.(2019) ^[58]^ | China | 92 | Cross-sectional | Moderate | N | 57% | 68.9±5.9 | 9±2.5 | Hypertension:30.2%, Diabetes:22.5%, Hyperlipidaemia:30.4%,Smoking:34.5% | MoCA: 21.7±4.3 | Philips | 3.0 T | ROI | Pearson |
| Wang et al.(2019) ^[59]^ | China | 120 | Cross-sectional | Low | N | 58% | 57.3±9.2 | N | Hypertension:32.9%, Diabetes:36.5%, Hyperlipidaemia:48.1% | MoCA: 24.4±5.9 | N | N | ROI | Pearson |
| Hui et al.(2021) ^[60]^ | China | 80 | Cross-sectional | Low | N | 58% | 64.1± 8.4 | 9.4±2.4 | N | MOCA: 25.8±3.8 | Siemens | 3.0 T | ROI | Pearson |
| Wang et al.(2021) ^[61]^ | China | 113 | Cross-sectional | Moderate | N | 78% | 65.1±7 | 10.5±2.6 | N | MOCA: 23.3±3.6 | GE | 3.0 T | ROI | Pearson |
| Lam et al.(2019) ^[62]^ | China/Hong Kong | 801 | cohort | High | WML volume: 6.35±10.8 | 29% | 71.8±5.1 | 7.87±4.92 | Hypertension:60.7%, Diabetes:22.6%, Hyperlipidaemia:30.2%,Smoking:22.2% | MoCA,: 23±6 | Philips | 3.0 T | TBSS | linear regression |
| Li et al.(2019) ^[63]^ | China | 71 | Cross-sectional | Moderate | MRI burden score≥1  (83.1%) | 54% | 69.8±8 | 9±3 | Hypertension:77.6%, Diabetes:32.4%, Hyperlipidaemia:16.9%,Smoking:33.8% | N | Shanghai United Film Company | 3.0 T | TBSS | Pearson |
| Reijmer YD et al.(2016) ^[64]^ | USA | 72 | Cross-sectional | Moderate | WML volume: 6.8±15.2 | 81% | 74.6±8.4 | 16.0±2.5 | N | MMSE: 28±4 | Siemens | 3.0 T | Network Reconstruction | Pearson |

**Category IV: CADASIL (N=7)**

| **Research ID** | **Country** | **Sample size** | **Study type** | **Quality** | **Severity** | **Male**  **(%)** | **Age** | **Education** | **Comorbidity** | **Cognition** | **Brand (MRI)** | **Magnet strength** | **Analysis** | **Statistics** |
| --- | --- | --- | --- | --- | --- | --- | --- | --- | --- | --- | --- | --- | --- | --- |
| O’Sullivan et al.(2004) ^[65]^ | UK | 31 | Cross-sectional | Low | N | N | 45.7±15.1 | N | N | MMSE: 29.2±1.0 | GE | 1.5 T | ROI | Pearson |
| Holtmannspo¨tter et al.(2005)^[66]^ | Germany | 64 | cohort | Moderate | N | 45% | 44.9±9.9 | N | N | SIDAM: 48.7±5.1 | N | 1.5 T | Histograms | Spearman |
| Ban et al.(2009) ^[67]^ | China | 36 | Cross-sectional | Moderate | Fazekas: 1.9±1.15 | 56% | 48.1±14.5 | N | N | MoCA: 21.2±8.5 | Siemens | 3.0 T | TBSS | Pearson |
| Zhang et al.(2021)^[68]^ | China | 23 | Cross-sectional | Low | N | 61% | 52.7±9.5 | 9.5±4.3 | N | MOCA: 24.5±3.7 | GE | 3.0 T | ROI | Pearson |
| Jacobsa et al.(2021) ^[69]^ | USA | 43 | Cross-sectional | Moderate | WM(%)：3.4±2.4% | 33% | 45.4±9.6 | 9.1±4.9 | Hypertension:25%, Diabetes:2%, Hyperlipidaemia:17.5%,Smoking:12% | MMSE:30 | Siemens | 3.0 T | ROI | Pearson |
| Yin et al.(2018) ^[70]^ | China | 29 | Cross-sectional | Moderate | WML volume: 64.1±31.7 | 59% | 48.4±7.9 | N | Hypertension:13.8%, Diabetes:3.4%, Hyperlipidaemia:20.7%,Smoking:20.7% | MMSE: 23.8±6.9 | GE | 3.0 T | ROI | multivariate linear regression |
| Viswanathan et al.(2010) ^[71]^ | France | 147 | cohort | Moderate | NAWM  (ml): 7.7±4.9 | 43% | 51.8±11.2 | High school: 62.1% | Hypertension:0.18.5%,Diabetes:2.7%, Hyperlipidaemia:43.2%,Smoking:49% | MMSE: 25.5±6.2 | GE | 1.5 T | Histograms | Multiple linear regression |

**Category V: Cerebral amyloid angiopathy (N=3)**

| **Research ID** | **Country** | **Sample size** | **Study type** | **Quality** | **Severity** | **Male**  **(%)** | **Age** | **Education** | **Comorbidity** | **Cognition** | **Brand (MRI)** | **Magnet strength** | **Analysis** | **Statistics** |
| --- | --- | --- | --- | --- | --- | --- | --- | --- | --- | --- | --- | --- | --- | --- |
| Viswanathan et al.(2008) ^[72]^ | France | 49 | cohort | Moderate | ICH volume: 41.3±24.8 | 55% | 73.3±7 | N | Hypertension72.1%, Diabetes:18.9%, Hyperlipidaemia:20.7% | N | GE | 1.5 T | N | Multivariate logistic regression |
| Reijmer et al.(2016) ^[73]^ | USA | 33 | cohort | High | ICH(%) :49% | 85% | 70.6±8.0 | 16±3 | N | MMSE: 27.9 ± 1.9 | Siemens | 1.5 T | Network Reconstruction | linear regression |
| Raposo et al.(2021) ^[74]^ | USA | 86 | case–control | Moderate | Normal WML: 0.33±0.62 | 62% | 73.7±7.7 | 16±2.6 | Hypertension:59.4%, Diabetes:10.2%, Hyperlipidaemia:62.6% | MMSE: 27.3±2.7 | Siemens | 3.0 T | TBSS | linear regression |

**Category VI : Cerebral microbleeds (N=2)**

| **Research ID** | **Country** | **Sample size** | **Study type** | **Quality** | **Severity** | **Male**  **(%)** | **Age** | **Education** | **Comorbidity** | **Cognition** | **Brand (MRI)** | **Magnet strength** | **Analysis** | **Statistics** |
| --- | --- | --- | --- | --- | --- | --- | --- | --- | --- | --- | --- | --- | --- | --- |
| Tan et al.(2020) ^[75]^ | China | 120 | Cross-sectional | Low | N | 67% | 61.6±8.6 | N | N | MOCA: 23.3±5.9 | GE | 3.0 T | ROI | linear regression |
| Patel et al.(2013) ^[76]^ | UK | 116 | Cross-sectional | Moderate | WML volume: 31.5±27.1 | 66% | 70.2±9.8 | N | Hypertension:89.2%, Diabetes:16.2%, Hyperlipidaemia:85.7%,Smoking:52.3% | N | GE | 1.5 T | Histograms | Spearman |

**Category VII: Fabry (N=1)**

| **Research ID** | **Country** | **Sample size** | **Study type** | **Quality** | **Severity** | **Male**  **(%)** | **Age** | **Education** | **Comorbidity** | **Cognition** | **Brand (MRI)** | **Magnet strength** | **Analysis** | **Statistics** |
| --- | --- | --- | --- | --- | --- | --- | --- | --- | --- | --- | --- | --- | --- | --- |
| Ulivi et al.(2020) ^[77]^ | UK | 50 | Cross-sectional | Moderate | MSSI: 17.5±8.6 | 48% | 41.6±36.8 | 15.8±3 | Hypertension:16.6%, Diabetes:0, Hyperlipidaemia:18%, Smoking:34.1% | N | Philips | 3.0 T | TBSS | multiple regression models |

References:

1. O'Sullivan M, Morris RG, Huckstep B, et al. Diffusion tensor MRI correlates with executive dysfunction in patients with ischemic leukoaraiosis. J Neurol Neurosurg Psychiatry 2004; 75:441-447.

2. Zhang XN，Zhang YT. Magnetic resonance imaging diffusion tensor imaging aging leukoaraiosis white matter . Journal of Clinical Radiology 2006; 25: 593-597.

3. Della NR, Foresti S, Pratesi A, et al. Whole-brain histogram and voxel-based analyses of diffusion tensor imaging in patients with leukoaraiosis: correlation with motor and cognitive impairment. AJNR Am J Neuroradiol 2007; 28:1313-1319.

4. Viana-Baptista M, Bugalho P, Jordão C, et al. Cognitive function correlates with frontal white matter apparent diffusion coefficients in patients with leukoaraiosis. JOURNAL OF NEUROLOGY 2008; 255:360-366.

5. Nitkunan A, Barrick TR, Charlton RA, et al. Multimodal MRI in cerebral small vessel disease: its relationship with cognition and sensitivity to change over time. STROKE 2008; 39:1999-2005.

6. Luan P, Wang JX, Lu BX, et al. Correlation between diffusion anisotropy of the white matter fibers and cognitive function in patients with leukoaraiosis. Journal of Southern Medical University 2009; 29: 1106-1110.

7. Qu CQ, Guo SG, Guo HZ, et al. Diffusion tensor imaging of elderly leukoaraiosis and its correlation with cognitive function. Chinese Medical Journal 2010; 90: 624-627.

8. Yuan TT. The study of leukoaraiosis by diffusion weighted imaging and diffusion tensor imaging. Jilin University 2010.

9. Li C, Ling X, Liu S, et al. Abnormalities of magnetic resonance spectroscopy and diffusion tensor imaging are correlated with executive dysfunction in patients with ischemic leukoaraiosis. JOURNAL OF CLINICAL NEUROSCIENCE 2012; 19:718-722.

10. Otsuka Y, Yamauchi H, Sawamoto N, et al. Diffuse tract damage in the hemispheric deep white matter may correlate with global cognitive impairment and callosal atrophy in patients with extensive leukoaraiosis. AJNR Am J Neuroradiol 2012; 33:726-732.

11. Zhang S. Application of diffusion tensor imaging in ischemic leukoaraiosis. J Modern Diagnosis & Treatment 2013; 17: 3841-3843.

12. Sun X, Liang Y, Wang J, et al. Early Frontal Structural and Functional Changes in Mild White Matter Lesions Relevant to Cognitive Decline. JOURNAL OF ALZHEIMERS DISEASE 2014; 40:123-550.

13. Pang R, Zhao J, Zhang YL, et al. The DTI in the evolution of vascular cognitive impairment mechanism based leukoaraiosis [J]. Journal of Practical Radiology 2014; 1071-1074.

14. Ding X, Wu J, Zhou Z, et al. Specific locations within the white matter and cortex are involved in the cognitive impairments associated with periventricular white matter lesions (PWMLs). BEHAVIOURAL BRAIN RESEARCH 2015; 289:9-18.

15. Pasi M, Salvadori E, Poggesi A, et al. White matter microstructural damage in small vessel disease is associated with Montreal cognitive assessment but not with mini mental state examination performances: vascular mild cognitive impairment Tuscany study. STROKE 2015; 46:262-264.

16. He YS, Jiang H, Li YX, et al. Association between Cognitive Impairment and Corpus Callosum Network Connectivity in Leukoaraiosis. Chinese Journal of Stroke 2016; 11: 449-454.

17. Yuan JL, Wang SK, Guo XJ, et al. Disconnections of Cortico-Subcortical Pathways Related to Cognitive Impairment in Patients with Leukoaraiosis: A Preliminary Diffusion Tensor Imaging Study. EUROPEAN NEUROLOGY 2017; 78:41-47.

18. Li SH, Li YC, Liang FR, et al. Study of the relationship between cognitive function of Leukoaraiosis and diffusion tensor imaging. Journal of Baotou Medical College 2017; 33:6-10.

19. He L, Zhao LQ, Shao HY, et al. Early cognitive impairment in patients with leukoaraiosis and its relation with diffusion tensor imaging. Chinese Journal of Neuromedicine, 2017; 16: 1235-1241.

20. Zhang W, Li J, Li X, Du Y. Diffusion Tensor Imaging of Cognitive Impairment Caused by Vascular Leukoencephalus Loosening. Neurology &amp; Neurophysiology 2019;10.

21. Wang L, Wang H. Application of cerebral magnetic resonance diffusion tensor imaging in elderly patients with vascular leukoencephalus cognitive impairment. Chinese Journal of Gerontology 2020; 40: 926-929.

22. Soriano-Raya JJ, Miralbell J, López-Cancio E, et al. Tract-specific fractional anisotropy predicts cognitive outcome in a community sample of middle-aged participants with white matter lesions. J Cereb Blood Flow Metab 2014; 34:861-869.

23. Zhong G, Zhang R, Jiaerken Y, et al. Better Correlation of Cognitive Function to White Matter Integrity than to Blood Supply in Subjects with Leukoaraiosis. Frontiers in Aging Neuroscience 2017; 9:185.

24. Wei N, Deng Y, Yao L, et al. A Neuroimaging Marker Based on Diffusion Tensor Imaging and Cognitive Impairment Due to Cerebral White Matter Lesions. Frontiers in Neurology 2019; 10:81.

25. Wang S, Jiaerken Y, Yu X, et al. Understanding the association between psychomotor processing speed and white matter hyperintensity: A comprehensive multi-modality MR imaging study. HUMAN BRAIN MAPPING 2020; 41:605-616.

26. Xing Y, Yang J, Zhou A, et al. White Matter Fractional Anisotropy Is a Superior Predictor for Cognitive Impairment Than Brain Volumes in Older Adults with Confluent White Matter Hyperintensities. Frontiers in Psychiatry 2021; 12:633811.

27. Wang Z, Bai L, Liu Q, et al. Corpus callosum integrity loss predicts cognitive impairment in Leukoaraiosis. Ann Clin Transl Neurol 2020; 7:2409-2420.

28. Chen HF, Huang LL, Li HY, et al. Microstructural disruption of the right inferior fronto-occipital and inferior longitudinal fasciculus contributes to WMH-related cognitive impairment. CNS Neuroscience & Therapeutics 2020; 26:576-588.

29. Zhou Y, Lin FC, Zhu J, et al. Whole brain diffusion tensor imaging histogram analysis in subcortical vascular dementia: A primary study. Chinese Journal of Medical Imaging Technology 2007; 23:479-481.

30. Yuan F, Liu YS, Zhao J, et al. Diffusion tensor imaging study of vascular dementia. CHINESE JOURNAL OF GERIATRIC HEART BRAIN AND VESSEL DISEASES 2009; 11:115-118.

31. Xu Q, Zhou Y, Li Y, et al. Diffusion Tensor Imaging Changes Correlate with Cognition Better than Conventional MRI Findings in Patients with Subcortical Ischemic Vascular Disease. DEMENTIA AND GERIATRIC COGNITIVE DISORDERS 2010; 30:317-326.

32. Zhang B, Wen CY, Wang L, et al. Functional MRI and cognition assessment in subcortical ischemic vascular disease. CHINESE JOURNAL OF INTERNAL MEDICINE 2011; 50: 411-415.

33. Dong YH. Correlation between cognitive impairment and neuroimaging and inflammatory markers in patients with subcortical ischemic vascular disease. Hebei Medical University 2011.

34. Lin L, Xue Y, Duan Q, et al. Microstructural White Matter Abnormalities and Cognitive Dysfunction in Subcortical Ischemic Vascular Disease: an Atlas-Based Diffusion Tensor Analysis Study. JOURNAL OF MOLECULAR NEUROSCIENCE 2015; 56:363-370.

35. Pan YJ, Zhou SH, Chen X, et al. Relationship between cognitive function and diffusion tensor imaging of white matter of subcortical ischemic vascular dementia patients. Journal of Clinical Neurology 2015; 28:5-8.

36. Chen H, Gao Y, Che C, et al. Diffusion Tensor Imaging with Tract-Based Spatial Statistics Reveals White Matter Abnormalities in Patients With Vascular Cognitive Impairment. Frontiers in Neuroanatomy 2018;12.

37. Liu X, Cheng R, Chen L, et al. Alterations of White Matter Integrity in Subcortical Ischemic Vascular Disease with and Without Cognitive Impairment: a TBSS Study. JOURNAL OF MOLECULAR NEUROSCIENCE 2019; 67:595-603.

38. Liao Z, Dang C, Li M, Bu Y, Han R, Jiang W. Microstructural damage of normal-appearing white matter in subcortical ischemic vascular dementia is associated with Montreal Cognitive Assessment scores. JOURNAL OF INTERNATIONAL MEDICAL RESEARCH 2019; 47:5723-5731.

39. Qiao Y, He X, Zhang J, et al. The Associations Between White Matter Disruptions and Cognitive Decline at the Early Stage of Subcortical Vascular Cognitive Impairment: A Case-Control Study. Frontiers in Aging Neuroscience 2021; 13:681208.

40. Guo SY, Chen B, Gen Y, et al. Application of Multimodal MRI in Vascular Cognitive Impairment Patients with Non-demented Cerebral ;Small Vessel Disease. Zhejiang Journal of Integrated Traditional Chinese and Western Medicine 2017;27:13-16.

41. D'Souza MM, Gorthi SP, Vadwala K, et al. Diffusion tensor tractography in cerebral small vessel disease: correlation with cognitive function. Neuroradiol J 2018; 31:83-89.

42. Zhao W, Lu D, Du J, et al. Diffusion Tensor Imaging Study of Cognitive Impairments Among Patients with Cerebral Small Vascular Disease. China Journal of Alzheimer's Disease and Related Disorders 2018; 1:193-198.

43. Li GQ, Guo CM, Zeng ZJ. Study on the Correlation between MRI Findings and Vascular Cognitive Impairment in Cerebral Small Vessel Disease. Chinese and Foreign Medical Research 2020;18:64-66.

44. Brandhofe A, Stratmann C, Schüre JR, et al. T2 relaxation time of the normal-appearing white matter is related to the cognitive status in cerebral small vessel disease. J Cereb Blood Flow Metab 2021; 41:1767-1777.

45. Lawrence AJ, Chung AW, Morris RG, Markus HS, Barrick TR. Structural network efficiency is associated with cognitive impairment in small-vessel disease. NEUROLOGY 2014; 83:304-311.

46. van Uden IW, van der Holst HM, Schaapsmeerders P, et al. Baseline white matter microstructural integrity is not related to cognitive decline after 5 years: The RUN DMC study. BBA Clin 2015; 4:108-114.

47. Wu YF, Wu WB, Liu QP, et al. Presence of lacunar infarctions is associated with the spatial navigation impairment in patients with mild cognitive impairment: a DTI study. Oncotarget 2016; 7:78310-78319.

48. Qin Q, Tang Y, Dou X, et al. Default mode network integrity changes contribute to cognitive deficits in subcortical vascular cognitive impairment, no dementia. Brain Imaging and Behavior 2021; 15:255-265.

49. Tu M, Lo C, Huang C, et al. Effectiveness of diffusion tensor imaging in differentiating early-stage subcortical ischemic vascular disease, Alzheimer's disease and normal ageing. PLoS One 2017;12.

50. Huang L, Chen X, Sun W, et al. Early Segmental White Matter Fascicle Microstructural Damage Predicts the Corresponding Cognitive Domain Impairment in Cerebral Small Vessel Disease Patients by Automated Fiber Quantification. Frontiers in Aging Neuroscience 2021;12.

51. Qiu Y, Yu L, Ge X, et al. Loss of Integrity of Corpus Callosum White Matter Hyperintensity Penumbra Predicts Cognitive Decline in Patients with Subcortical Vascular Mild Cognitive Impairment. Frontiers in Aging Neuroscience 2021; 13:605900.

52. Du J, Zhu H, Yu L, et al. Multi-Dimensional Diffusion Tensor Imaging Biomarkers for Cognitive Decline from the Preclinical Stage: A Study of Post-stroke Small Vessel Disease. Frontiers in Neurology 2021; 12:687959.

53. Chen DQ, Deng QP, Pan DJ. Relationship between functional features of multimode MR scan and cognitive impairment in patients with cerebral small vessel disease. Chinese Journal of Rehabilitation 2014:173-175.

54. Dai LW, Li H, Jin CL. The study of the correlation between diffusion tensor imaging and cognitive impairment in cerebral small vessel disease. Journal of Practical Radiology 2015:646-649.

55. Liu JP, Zhao H, Gao MY et al. The Study of Correlation Between Diffusion Tensor Imaging and Cognitive Impairment in Cerebral Small Vessel Disease. Chinese journal of CT and MRI 2016;14:90-92, 95.

56. Hu JQ, Shen S. Relationship between the imaging manifestations of cerebral small vessel disease and cognitive dysfunction. China Modern Doctor 2017; 55:94-97.

57. Liu D, Li K, Ma X, et al. Correlations Between the Microstructural Changes of the Medial Temporal Cortex and Mild Cognitive Impairment in Patients With Cerebral Small Vascular Disease (cSVD): A Diffusion Kurtosis Imaging Study. Frontiers in Neurology 2019; 10:1378.

58. Zhao J, Chang WL, Song WL, et al. Relationship between Cognitive Impairment and White Matter Integrity, Cerebral Blood Flow Perfusion in Patients with Cerebral Small Vessel Disease. Neural Injury and Functional Reconstruction 2019; 14:433-436.

59. Wang ZP, Gao ZL, Wei MJ. Magnetic resonance imaging findings of cerebral small vessel disease and vascular cognitive impairment. Chinese Journal for Clinicians 2019; 47:814-817.

60. Liu H, Liu D, Li K, et al. Microstructural changes in the cingulate gyrus of patients with mild cognitive impairment induced by cerebral small vessel disease. NEUROLOGICAL RESEARCH 2021; 43:659-667.

61. Wang Y, Lu P, Zhan Y, et al. The Contribution of White Matter Diffusion and Cortical Perfusion Pathology to Vascular Cognitive Impairment: A Multimode Imaging-Based Machine Learning Study. Frontiers in Aging Neuroscience 2021; 13:687001.

62. Lam B, Leung KT, Yiu B, et al. Peak width of skeletonized mean diffusivity and its association with age-related cognitive alterations and vascular risk factors. Alzheimers Dement (Amst) 2019; 11:721-729.

63. Li YF, Zhao Z. Cognitive impairment in patients with cerebral small vessel disease: comparison of MRI total burden and diffusion tensor tract-based spatial statistics. International Journal of Cerebrovascular Diseases 2019; 27:736-742.

64. Reijmer YD, Fotiadis P, Piantoni G, et al. Small vessel disease and cognitive impairment: The relevance of central network connections. HUMAN BRAIN MAPPING 2016; 37:2446-2454.

65. O'Sullivan M, Singhal S, Charlton R, Markus HS. Diffusion tensor imaging of thalamus correlates with cognition in CADASIL without dementia. NEUROLOGY 2004; 62:702-707.

66. Holtmannspötter M, Peters N, Opherk C, et al. Diffusion magnetic resonance histograms as a surrogate marker and predictor of disease progression in CADASIL: a two-year follow-up study. STROKE 2005; 36:2559-2565.

67. Ban S, Wang H, Wang M, et al. Diffuse Tract Damage in CADASIL Is Correlated with Global Cognitive Impairment. EUROPEAN NEUROLOGY 2019;81: 294-301.

68. Zhang Q, Wang D, Wu S, et al. Diffuse Tract Damage Correlates with Global Cognitive Impairment in Cerebral Autosomal Dominant Arteriopathy With Subcortical Infarcts and Leukoencephalopathy: A Tract-Based Spatial Statistics Study. JOURNAL OF COMPUTER ASSISTED TOMOGRAPHY 2021; 45:285-293.

69. Jacobs H, Schoemaker D, Torrico-Teave H, et al. Specific Abnormalities in White Matter Pathways as Interface to Small Vessels Disease and Cognition in Cerebral Autosomal Dominant Arteriopathy with Subcortical Infarcts and Leukoencephalopathy Individuals. Brain Connect 2021.

70. Yin X, Zhou Y, Yan S, Lou M. Effects of Cerebral Blood Flow and White Matter Integrity on Cognition in CADASIL Patients. Frontiers in Psychiatry 2018; 9:741.

71. Viswanathan A, Godin O, Jouvent E, et al. Impact of MRI markers in subcortical vascular dementia: a multi-modal analysis in CADASIL. NEUROBIOLOGY OF AGING 2010; 31:1629-1636.

72. Viswanathan A, Patel P, Rahman R, et al. Tissue microstructural changes are independently associated with cognitive impairment in cerebral amyloid angiopathy. STROKE 2008; 39:1988-1992.

73. Reijmer YD, Fotiadis P, Riley GA, et al. Progression of Brain Network Alterations in Cerebral Amyloid Angiopathy. STROKE 2016; 47:2470-2475.

74. Raposo N, Zanon ZM, Schoemaker D, et al. Peak Width of Skeletonized Mean Diffusivity as Neuroimaging Biomarker in Cerebral Amyloid Angiopathy. AJNR Am J Neuroradiol 2021; 42:875-881.

75. Tan QJ, Liu YH, Yang JW, et al. Association study on neuroimaging features and cognitive impairment of patients with cerebral microbleeds. China Medical Herald 2020;17:89-92.

76. Patel B, Lawrence AJ, Chung AW, et al. Cerebral microbleeds and cognition in patients with symptomatic small vessel disease. STROKE 2013; 44:356-361.

77. Ulivi L, Kanber B, Prados F, et al. White matter integrity correlates with cognition and disease severity in Fabry disease. BRAIN 2021; 143:3331-3342.
