# Supplementary material for: Association between white matter alterations and domain-specific cognitive impairment in cerebral small vessel disease: A meta-analysis of diffusion tensor imaging
Source: Front Aging Neurosci. 2022 Nov 22;14:1019088. doi: 10.3389/fnagi.2022.1019088 (PMC9722766; doi:10.3389/fnagi.2022.1019088)
Supplement: Supplementary file 2 [file Table_2.docx]

Table S2. summary of univariate meta-regression analysis

| **Heterogeneity variables** | **OR (95%CI)** | ***p*** | **R^2^** | **I^2^** |
| --- | --- | --- | --- | --- |
| **FA - Overall** | | | | |
| Sample size | 0.86 (0.82, 0.93) | **0.000** | 8.42% | 73.36% |
| Aetiology | 1.09 (1, 1.19) | **0.000** | 1.89% | 75.38% |
| Magnet strength | 1.18 (1.1, 1.27) | **0.000** | 8.15% | 74.13% |
| Study type | 0.9 (0.81, 1) | **0.054** | 2.91% | 71.17% |
| Quality of study | 0.95(0.91, 0.99) | **0.032** | 3.87% | 71.31% |
| **MD/ADC- Overall** | | | | |
| Sample size | 1.13 (1.06, 1.2) | **0.000** | 6.92% | 74.35% |
| Aetiology | 0.9 (0.82, 0.98) | **0.013** | 3.42% | 75.10% |
| Magnet strength | 0.99 (0.92, 1.05) | 0.648 | -0.64% | 76.32% |
| Study type | 0.96 (0.89, 1.03) | 0.241 | -0.41% | 75.04% |
| Quality of study | 1.09 (1.03, 1.15) | **0.002** | 6.58% | 71.69% |
| **FA- Corpus Callosum** | | | | |
| Sample size | 0.69 (0.4, 1.19) | 0.162 | 9.70% | 88.59% |
| Aetiology | 1.85 (0.83, 4.12) | 0.123 | 14.63% | 87.73% |
| Magnet strength | 1.7 (0.54, 5.36) | 0.340 | -0.34% | 89.71% |
| Study type | Cross-sectional study | | | |
| Quality of study | 1.4 (0.8, 2.47) | 0.216 | 5.02% | 89.53% |
| **FA - Frontal Lobe** | | | | |
| Sample size | 0.87 (0.57, 1.34) | 0.497 | -6.44% | 80.58% |
| Aetiology | Arteriosclerosis | | | |
| Magnet strength | 1.34 (0.78, 2.32) | 0.260 | 5.56% | 78.37% |
| Study type | Cross-sectional study | | | |
| Quality of study | 1.36 (0.87, 2.13) | 0.157 | 7.83% | 80.18% |
| **MD/ADC - Centrum Semiovale** | | | | |
| Sample size | 0.88 (0.53, 1.49) | 0.605 | -7.92% | 86.84% |
| Aetiology | 1.1 (0.47, 2.55) | 0.804 | -10.03% | 87.04% |
| Magnet strength | 1.25 (0.77, 2.01) | 0.319 | 2.55% | 86.13% |
| Study type | Cross-sectional study | | | |
| Quality of study | 1.23 (0.76, 1.97) | 0.359 | -0.34% | 85.49% |
| **MD/ADC - Corpus Callosum** | | | | |
| Sample size | 1.41 (0.73, 2.74) | 0.264 | 5.12% | 83.38% |
| Aetiology | 0.55 (0.17, 1.73) | 0.260 | 8.46% | 83.29% |
| Magnet strength | 0.75 (0.23, 2.45) | 0.587 | -10.85% | 85.72% |
| Study type | Cross-sectional study | | | |
| Quality of study | 0.79 (0.4, 1.57) | 0.454 | -6.58% | 85.46% |
| **MD/ADC - Frontal Lobe** | | | | |
| Sample size | 1.17 (0.71, 1.95) | 0.486 | -6.32% | 83.14% |
| Aetiology | Arteriosclerosis | | | |
| Magnet strength | 0.94 (0.61, 1.45) | 0.753 | -11.42% | 80.48% |
| Study type | Cross-sectional study | | | |
| Quality of study | 1.21 (0.81, 1.79) | 0.312 | -0.21% | 82.13% |

Table S3. Subgroup analyses examining impact of specific methodological variables on the effects sizes (FA and MD/ADC data)

| **Variables** | | **Correlation Coefficients** | ***p*** | **I^2^** |
| --- | --- | --- | --- | --- |
| **FA - Overall** | | | | |
| Sample size | ≥50 | 0.435 (0.394, 0.473) | 0.000 | 68.2% |
|  | < 50 | 0.341 (0.296, 0.350) | 0.000 | 76.6% |
| Aetiology | Arteriosclerosis | 0.359 (0.334, 0.383) | 0.000 | 76.3% |
|  | Genetic | 0.433 (0.359, 0.503) | 0.000 | 68.9% |
| Magnet strength | 1.5 Tesla | 0.243 (0.198, 0.286) | 0.000 | 60.4% |
|  | 3 Tesla | 0.399 (0.372, 0.424) | 0.000 | 76.2% |
| Quality of study | High quality | 0.199 (0.147, 0.251) | 0.000 | 62.5% |
|  | Moderate quality | 0.384 (0.352, 0.414) | 0.000 | 73.5% |
|  | Low quality | 0.382 (0.343, 0.419) | 0.000 | 76.8% |
| Study type | Cross-sectional | 0.378 (0.354, 0.401) | 0.000 | 69.9% |
|  | Cohort | 0.131 (0.083, 0.178) | 0.000 | 65.9% |
|  | Case–control | 0.652 (0.504, 0.763) | 0.000 | 0% |
| Overall | | 0.370 (0.346, 0.393) | 0.000 | 76% |
| **MD/ADC- Overall** | | | | |
| Sample size | ≥50 | -0.398 (-0.448, -0.346) | 0.000 | 69.2% |
|  | < 50 | -0.288 (-0.315, -0.260) | 0.000 | 76.7% |
| Aetiology | Arteriosclerosis | -0.309 (-0.334, -0.283) | 0.000 | 74.1% |
|  | Genetic | -0.404 (-0.490, -0.311) | 0.000 | 79.2% |
| Quality of study | High quality | -0.156 (-0.206, -0.104) | 0.000 | 74% |
|  | Moderate quality | -0.322 (-0.350, -0.294) | 0.000 | 69.6% |
|  | Low quality | -0.371 (-0.431, -0.310) | 0.000 | 75% |
| Overall | | -0.323 (-0.348, -0.298) | 0.000 | 76.1% |

Table S4. Summary findings for regions and cognitive domains with large (r ≥ 0.5) correlations based on the findings of more than one study

| **Regions** | **DTI** | **Correlation Coefficients** | | | |
| --- | --- | --- | --- | --- | --- |
|  |  | General cognition | Executive function | Memory | Attention |
| **CG** | FA | 0.591 |  |  | 0.532 |
|  | MD/ADC | -0.526 |  |  |  |
| **CC** | FA |  |  |  | 0.538 |
|  | MD/ADC |  |  | -0.730 |  |
| **CP** | FA | 0.584 |  |  |  |
| **CR** | FA | 0.543 |  |  |  |
| **EC** | FA | 0.662 |  |  |  |
| **FL** | FA | 0.614 | 0.523 |  | 0.732 |
| **FOF** | FA |  | 0.509 |  |  |
| **FOR** | FA | 0.543 |  |  |  |
| **IC** | FA | 0.597 |  |  |  |
| **TR** | FA | 0.571 |  |  |  |
| **NAWM** | MD/ADC | -0.546 |  |  |  |
| **WBWM** | MD/ADC | -0.505 |  |  |  |

Table S5: Summary of abbreviating words in the article

| **Abbreviating Words** | |
| --- | --- |
| anterior periventricular | APV |
| attention | AT |
| cerebral amyloid angiopathy | CAA |
| cerebral autosomal dominant arteriopathy with subcortical infarcts and leukoencephalopathy | CADASIL |
| corpus callosum | CC |
| central connections | CCS |
| cerebellum | CER |
| concept formation and reasoning | CFR |
| cingulate gyrus | CG |
| cerebral microbleeding | CMB |
| construction and motor performance | CMP |
| caudate nucleus | CN |
| corona radiata | CR |
| centrum semiovale | CS |
| cerebral small vessel disease | CSVD |
| corticospinal tract | CT |
| cerebral peduncle | CP |
| diffusion tensor imaging | DTI |
| external capsule | EC |
| executive function | EF |
| forceps major | FMA |
| forceps minor | FMI |
| fornix | FOR |
| fronto-occipital fasciculus | FOF |
| frontal lobe | FL |
| general cognition | GC |
| globus pallidus | GP |
| hemispheric deep white matter | HDWM |
| hippocampus | HIP |
| inferior longitudinal fasciculus | ILF |
| internal capsule | IC |
| medial lemniscus | ML |
| memory | ME |
| normal appearing white matter | NAWM |
| non-central connections | NCC |
| occipital lobe | OL |
| putamen | PUT |
| parahippocampal gyrus | PHG |
| parietal lobe | PL |
| periventricular | PV |
| posterior periventricular | PPV |
| processing speed and working memory | PSWM |
| subcortical ischemic vascular disease | SIVD |
| superior longitudinal fasciculus | SLF |
| sagittal stratum | SS |
| temporal lobe | TL |
| thalamus | THA |
| thalamic radiation | TR |
| uncinate fasciculus | UF |
| verbal skills | VS |
| whole brain white matter | WBWM |
| white matter hyperintensities | WMH |
| white matter lesion | WML |
