# Supplementary figures and images for: Association between white matter alterations and domain-specific cognitive impairment in cerebral small vessel disease: A meta-analysis of diffusion tensor imaging
Source: Front Aging Neurosci. 2022 Nov 22;14:1019088. doi: 10.3389/fnagi.2022.1019088 (PMC9722766; doi:10.3389/fnagi.2022.1019088)

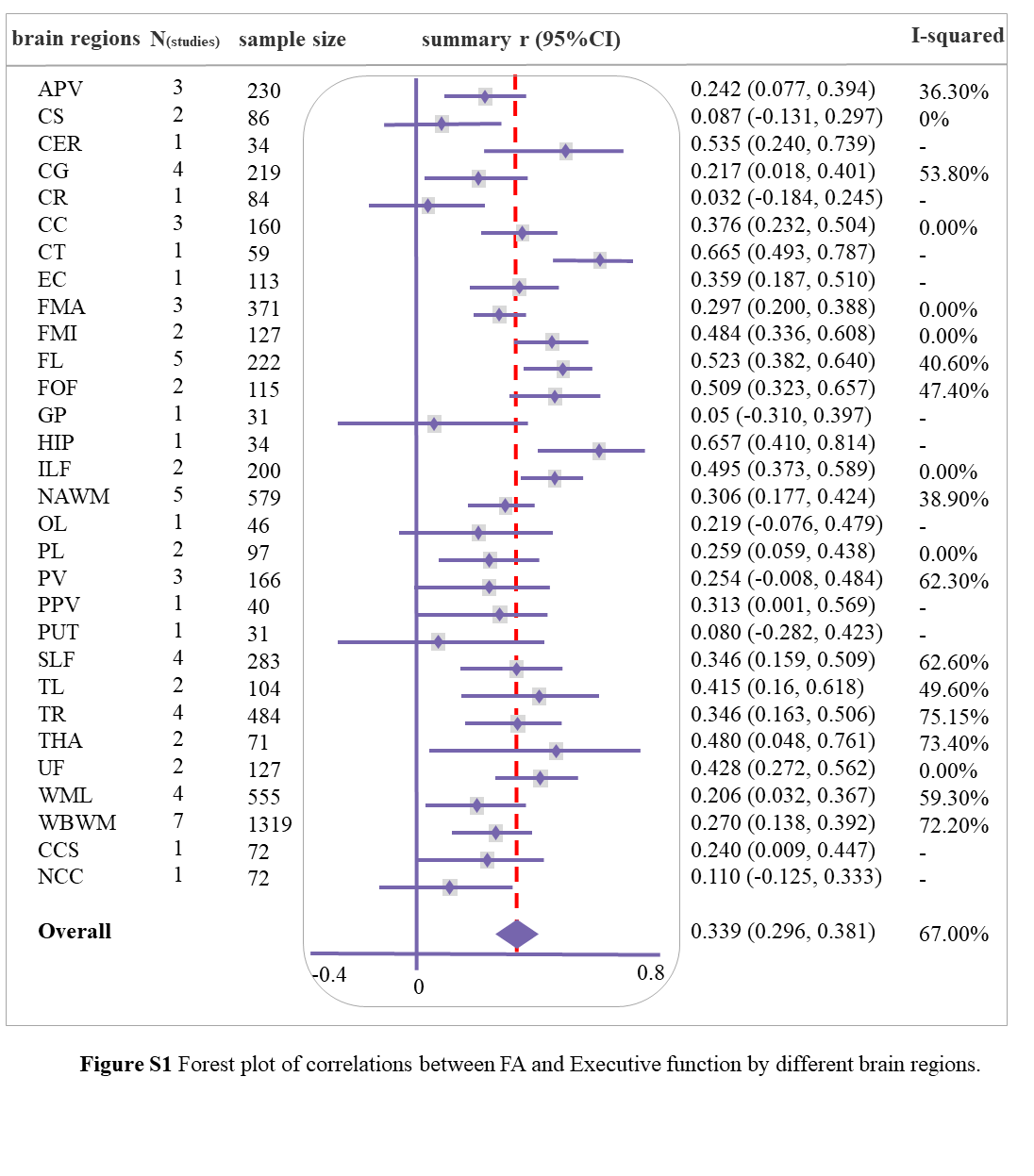

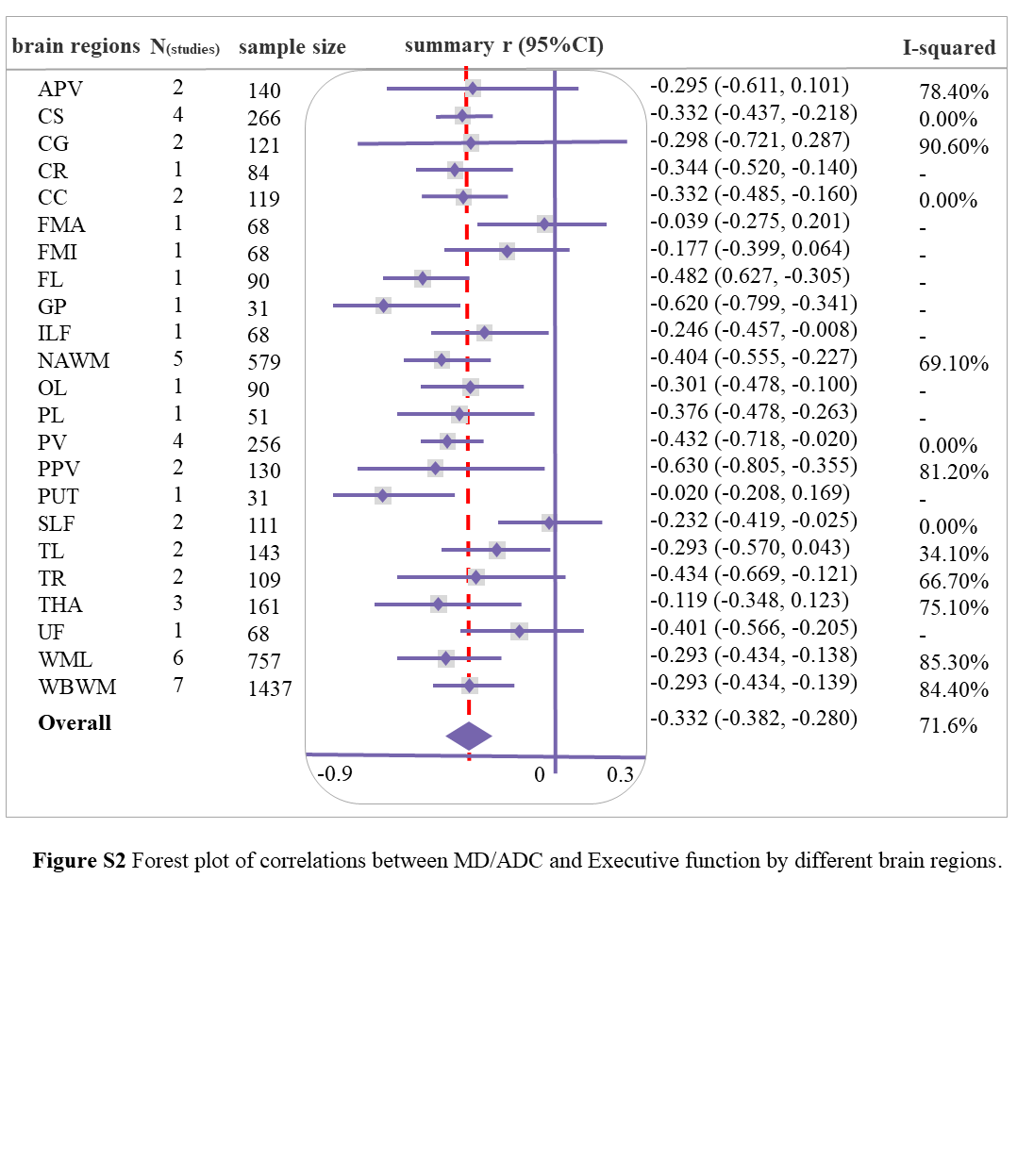

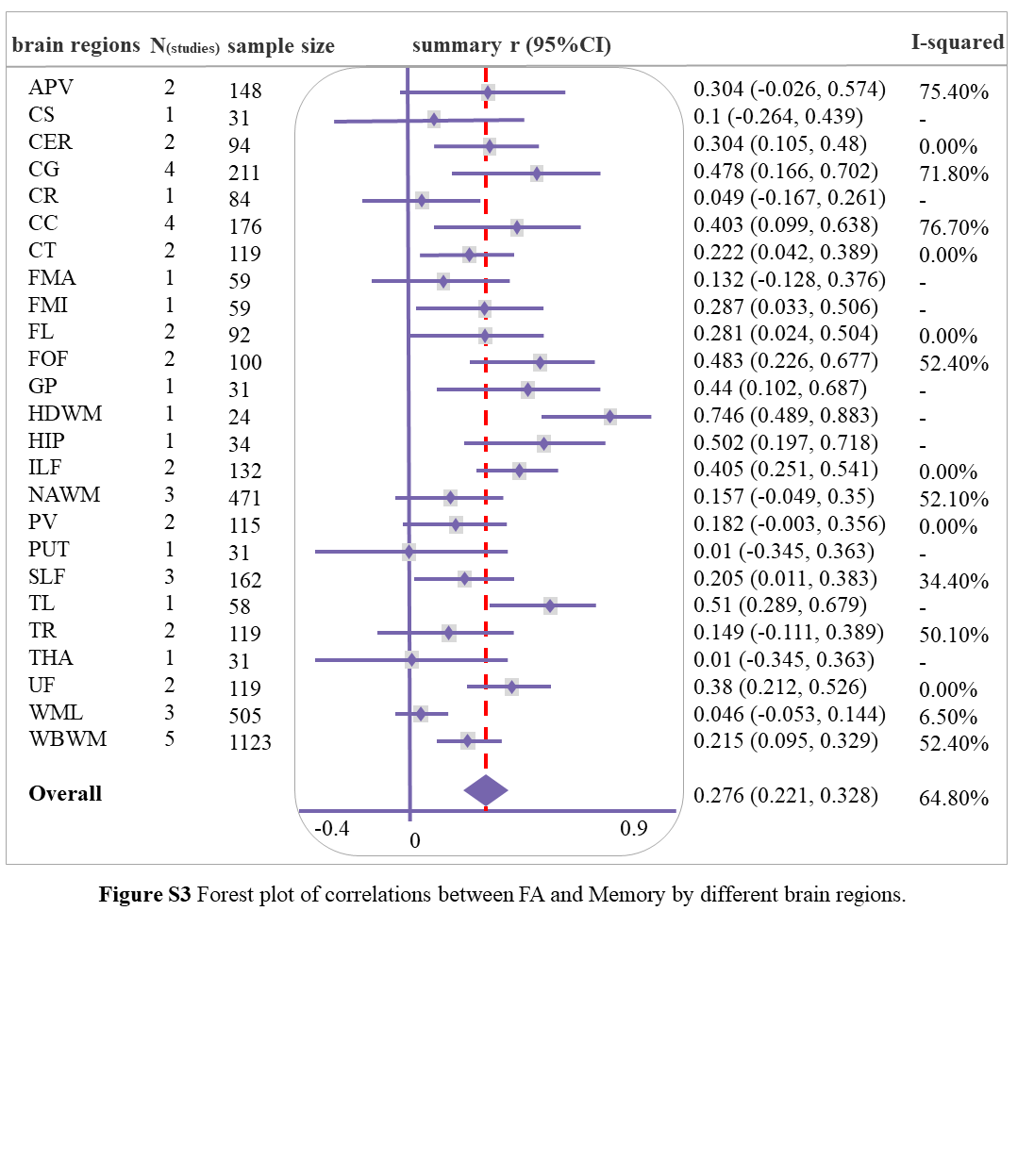

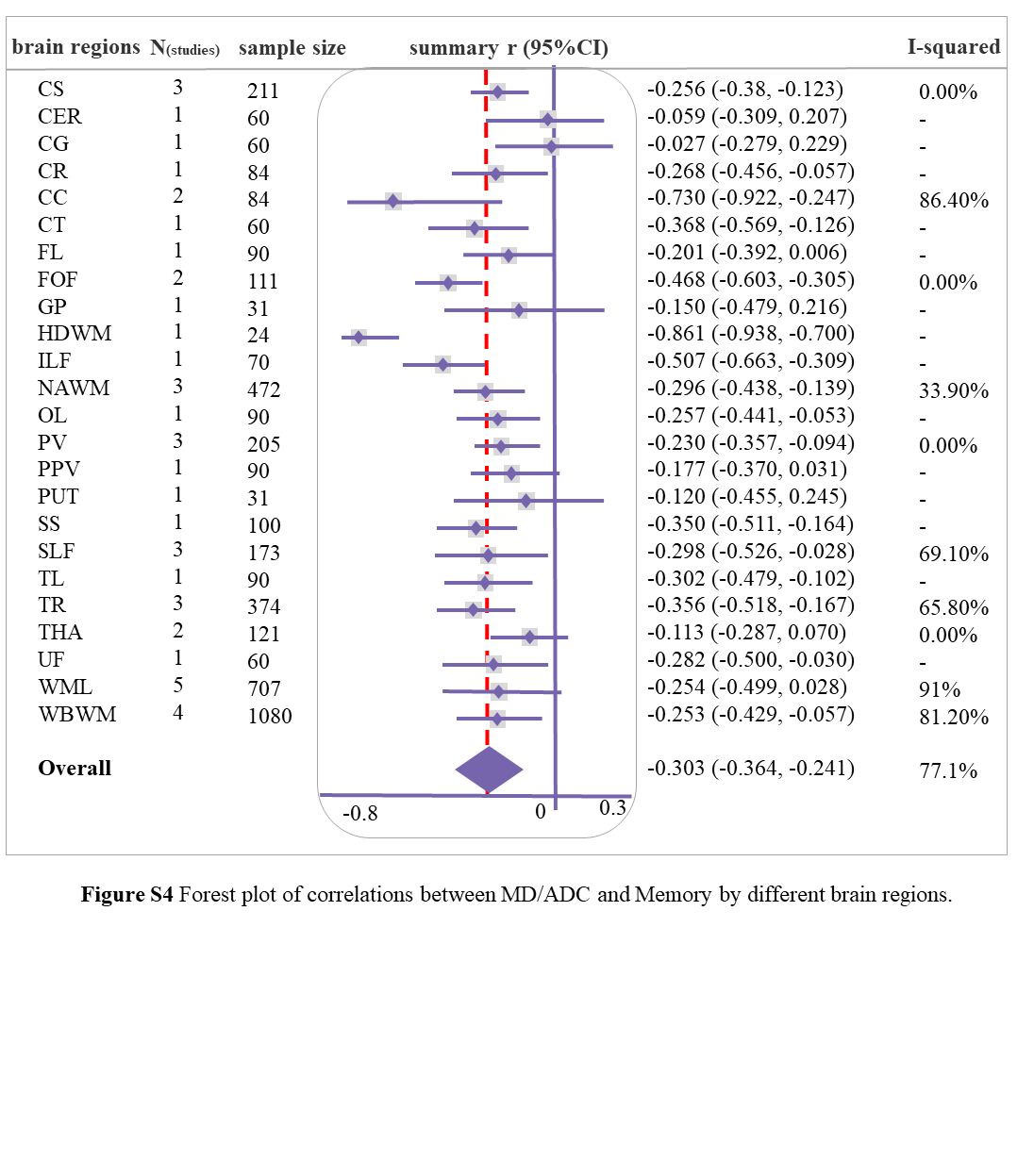

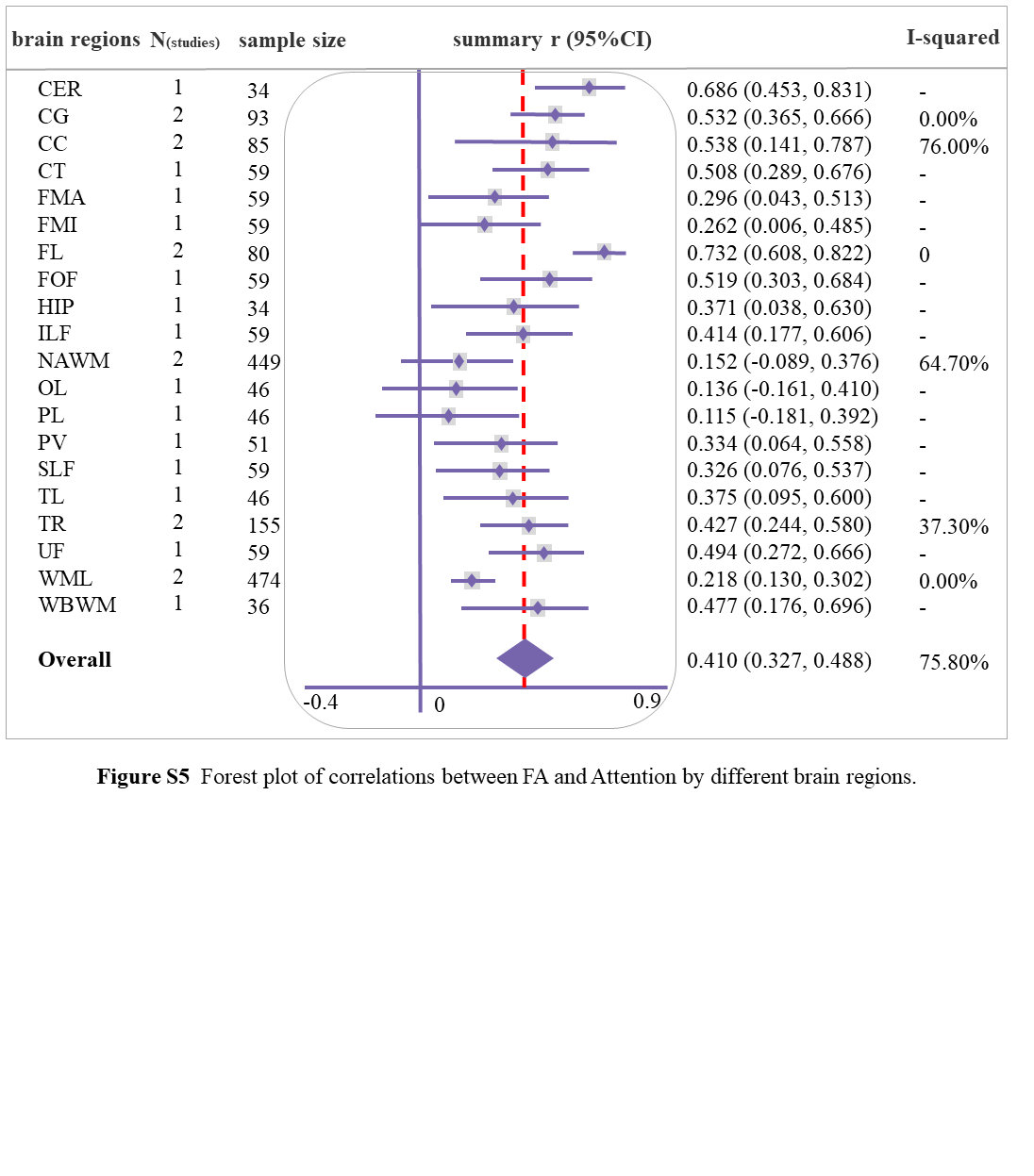


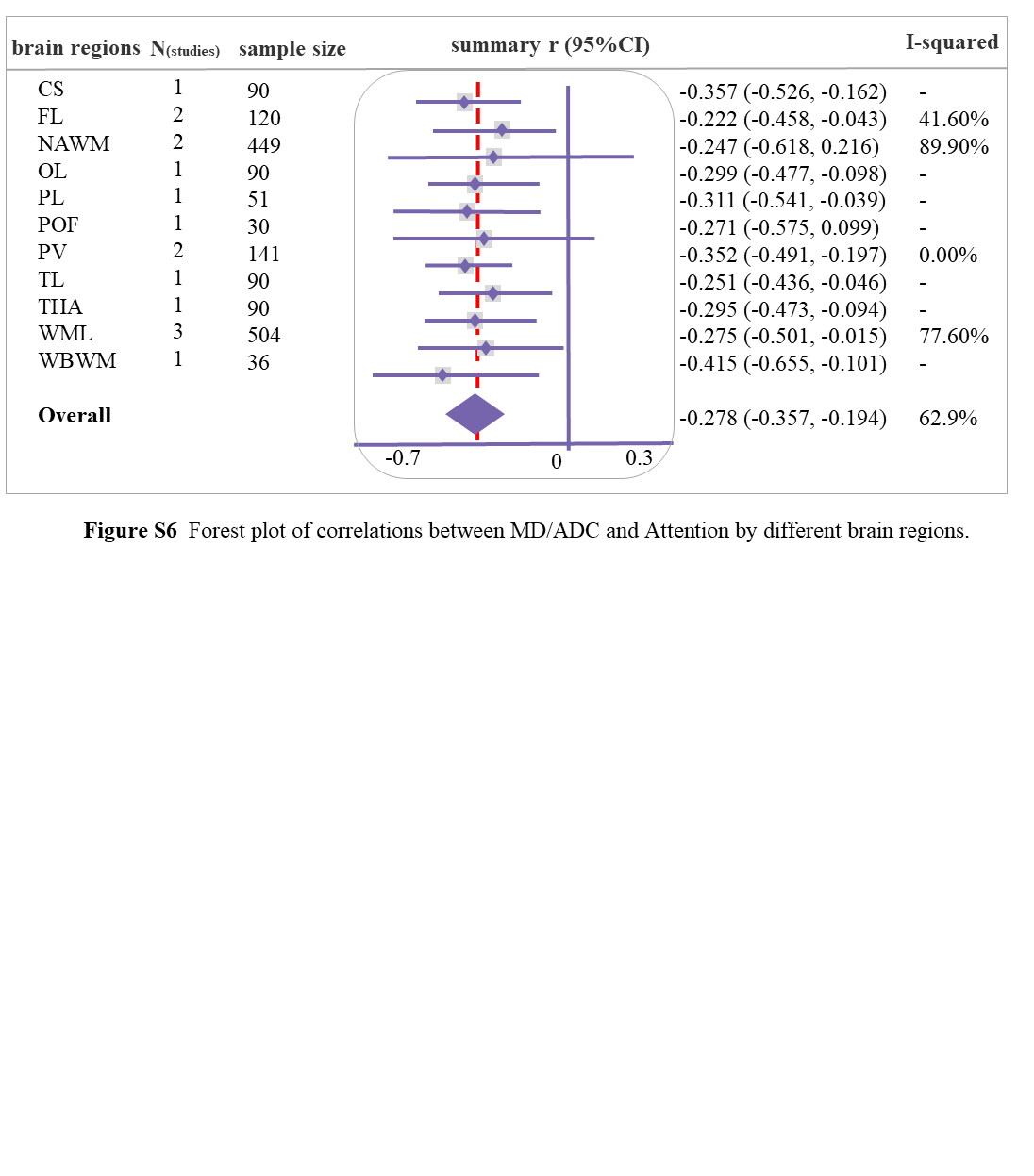

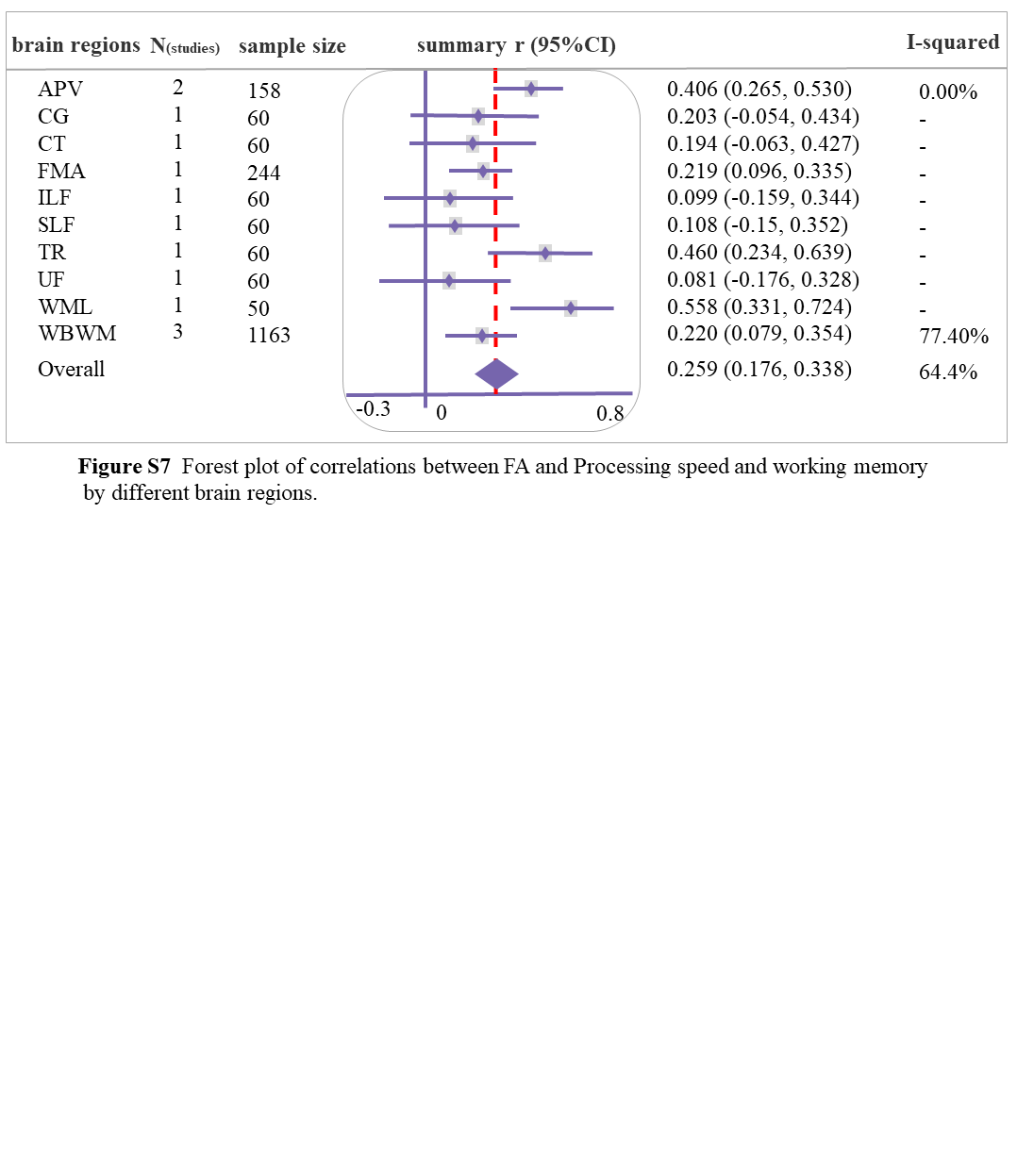


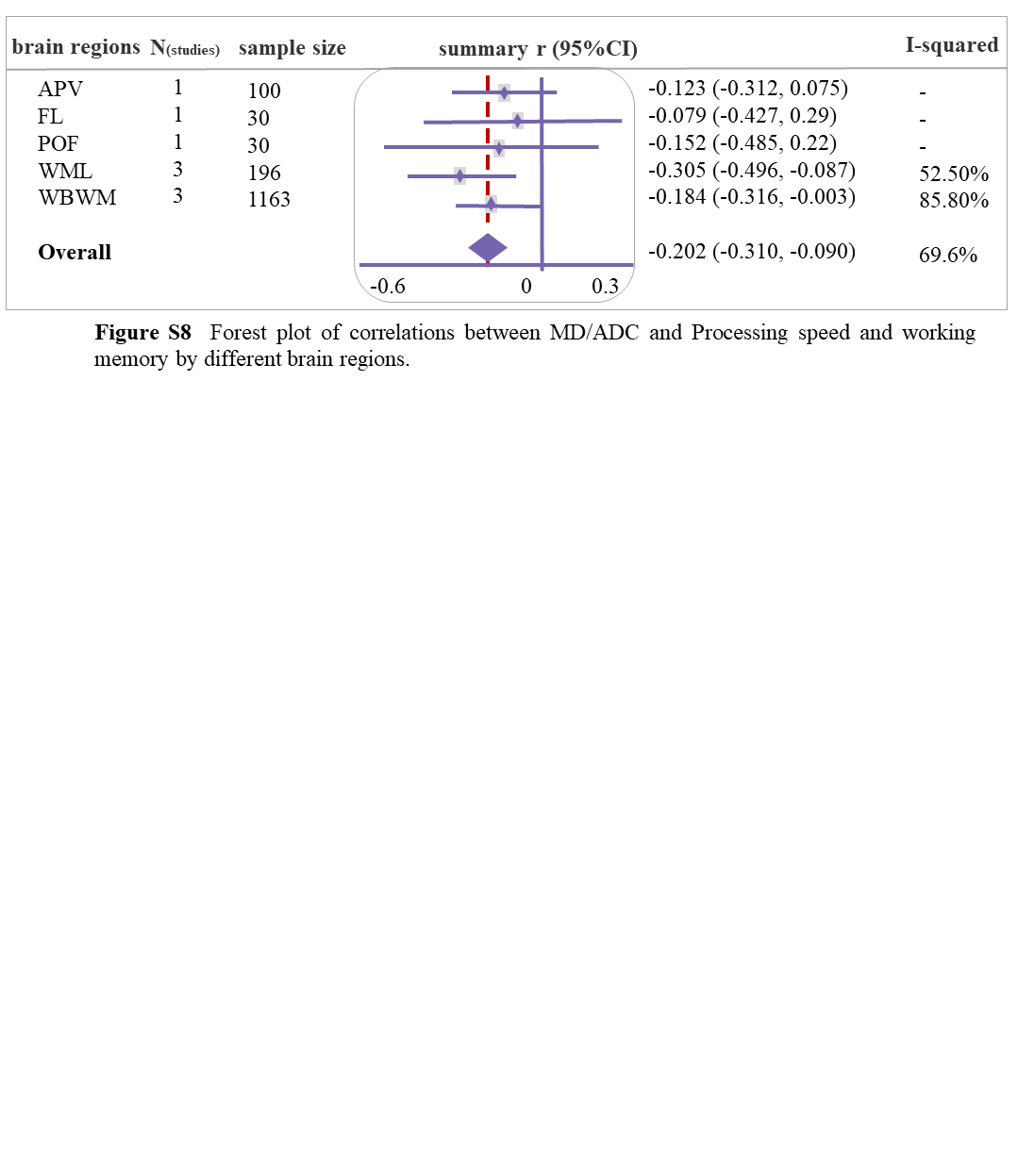

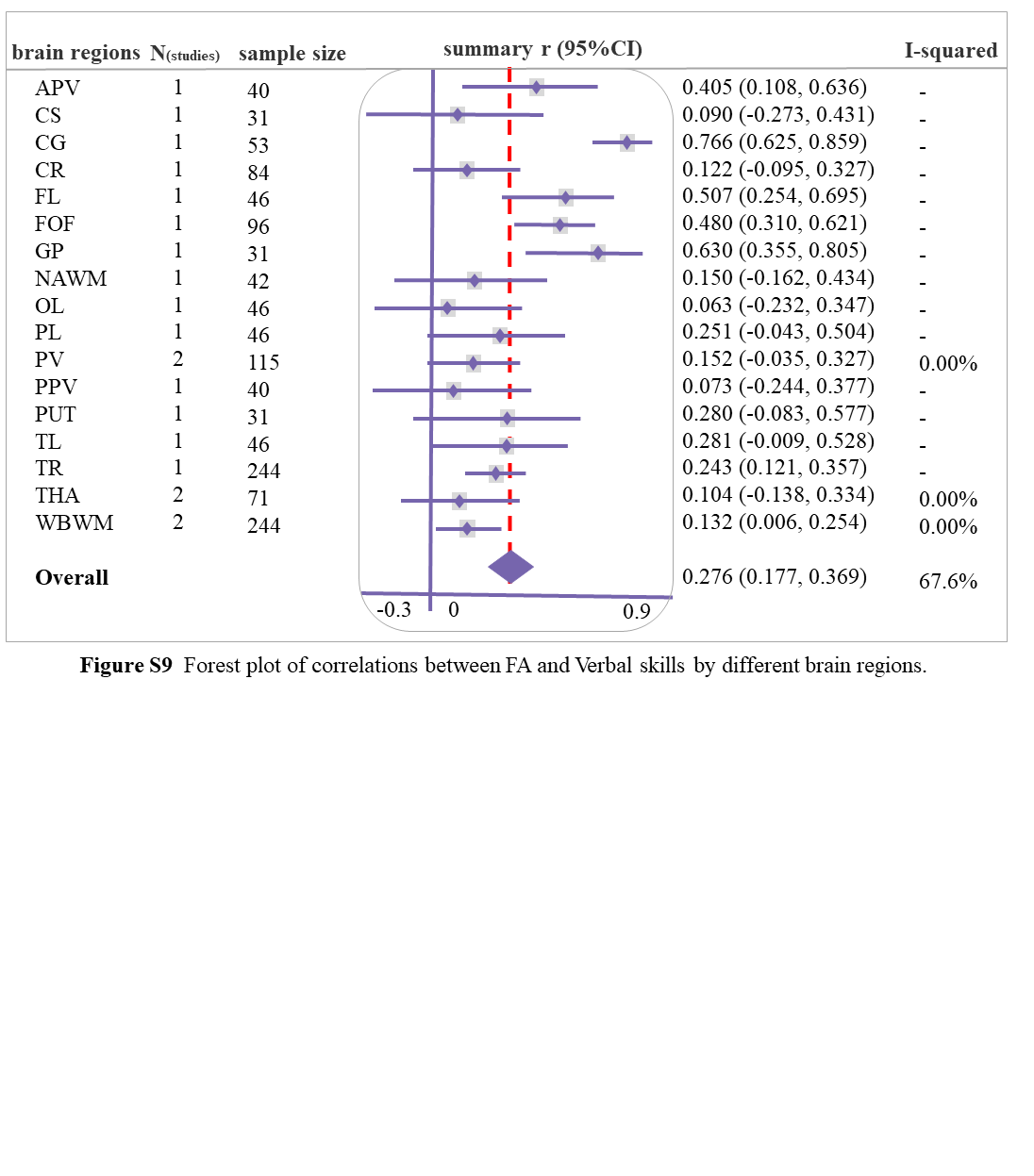


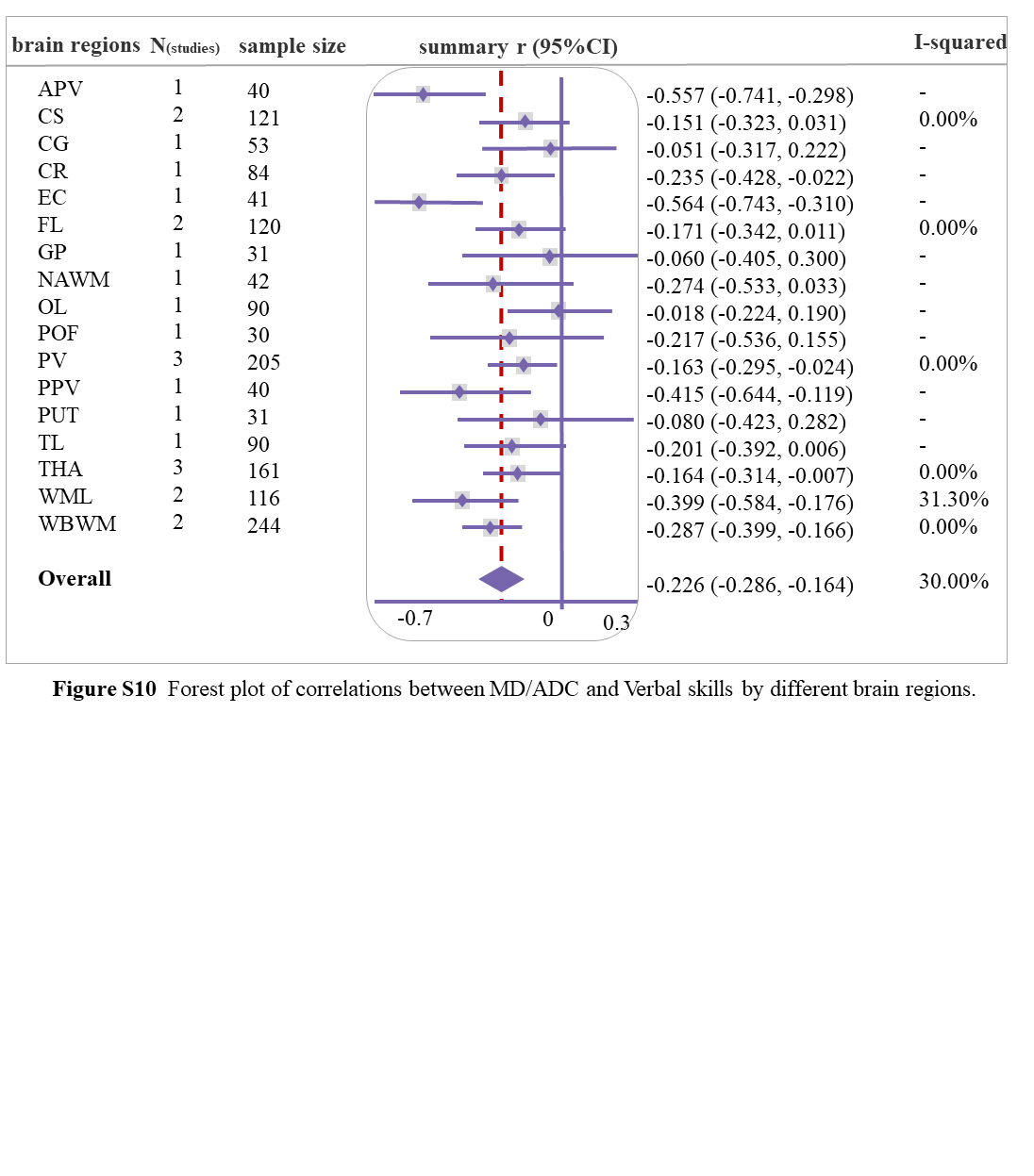

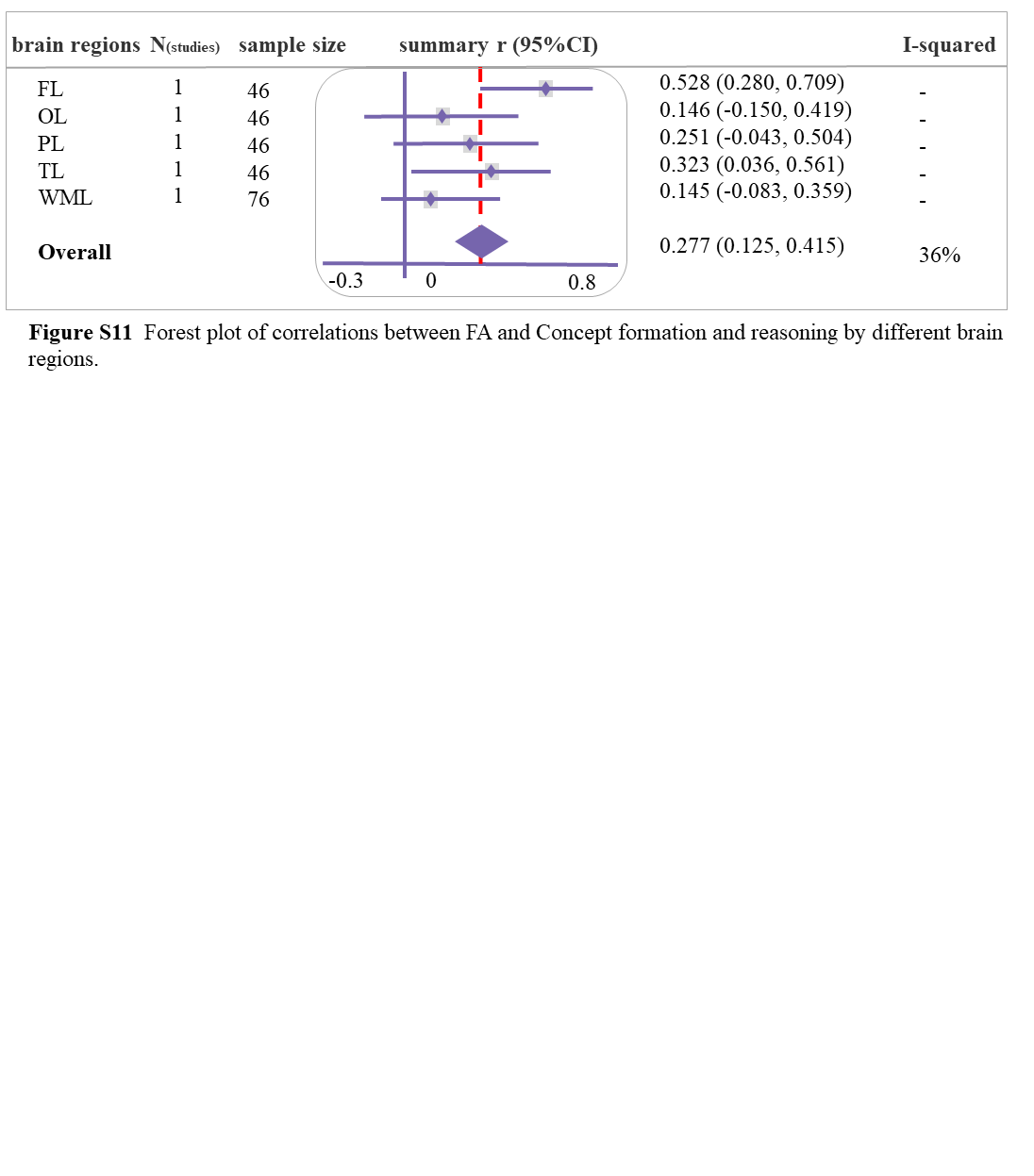


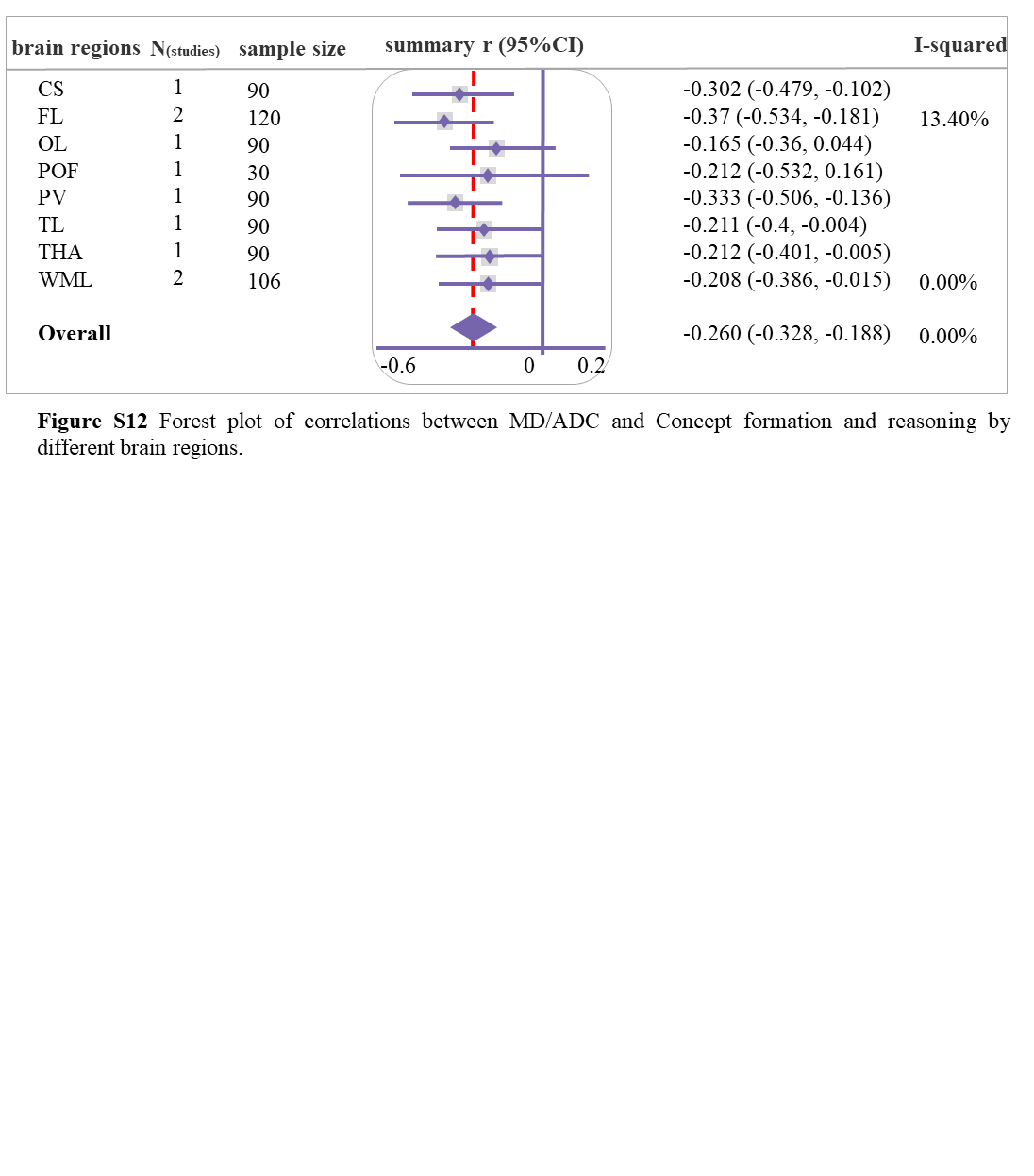


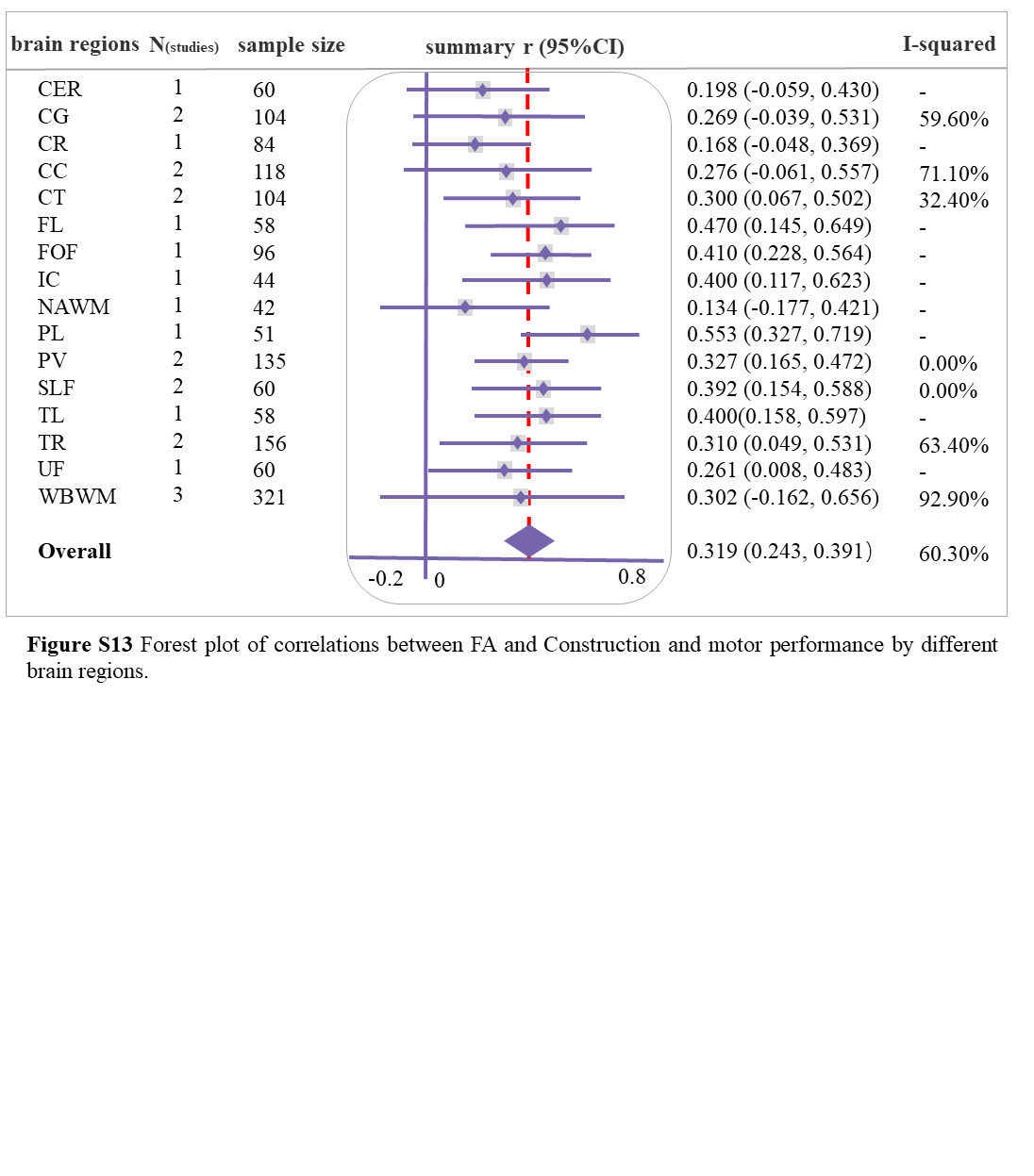


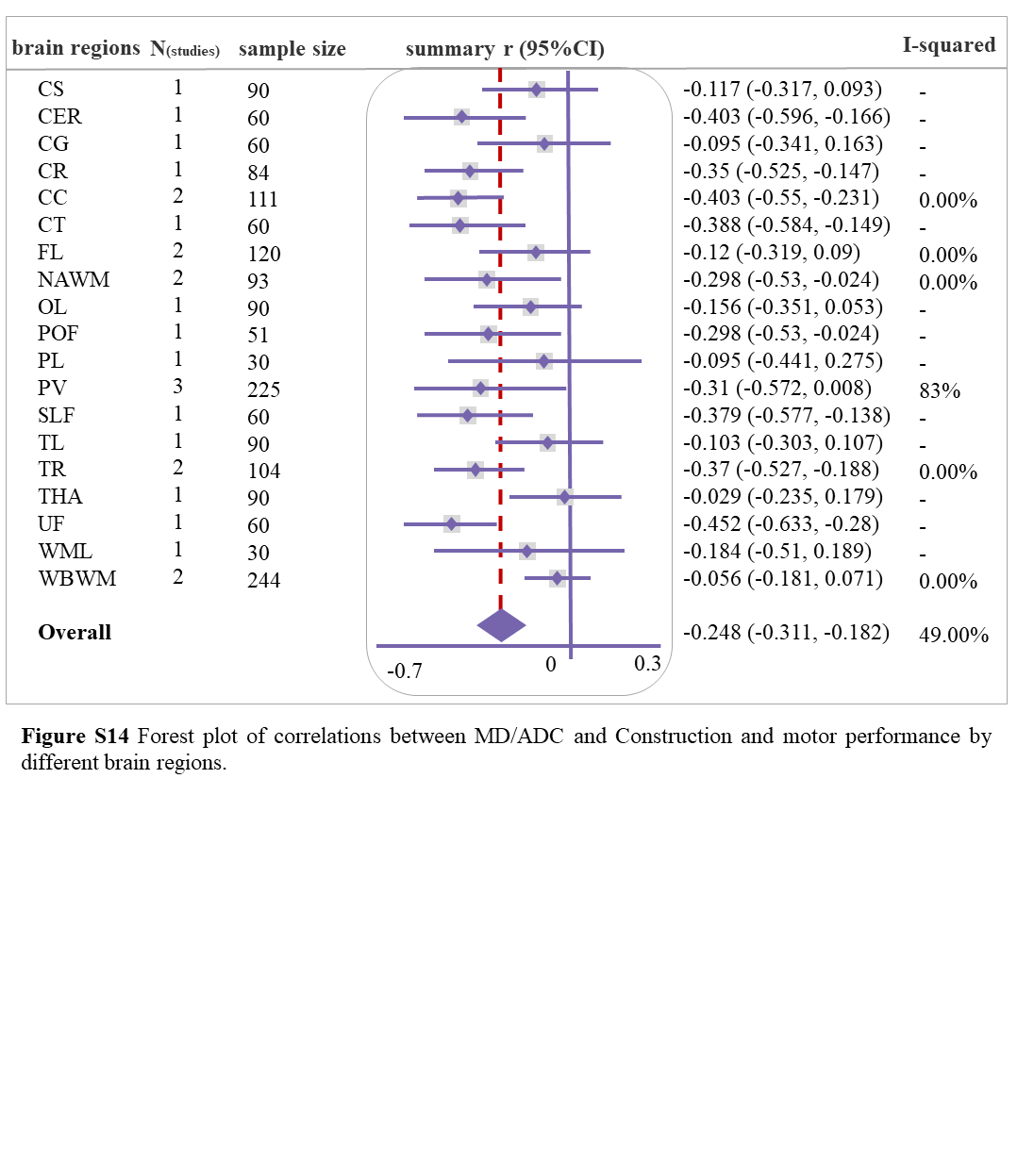


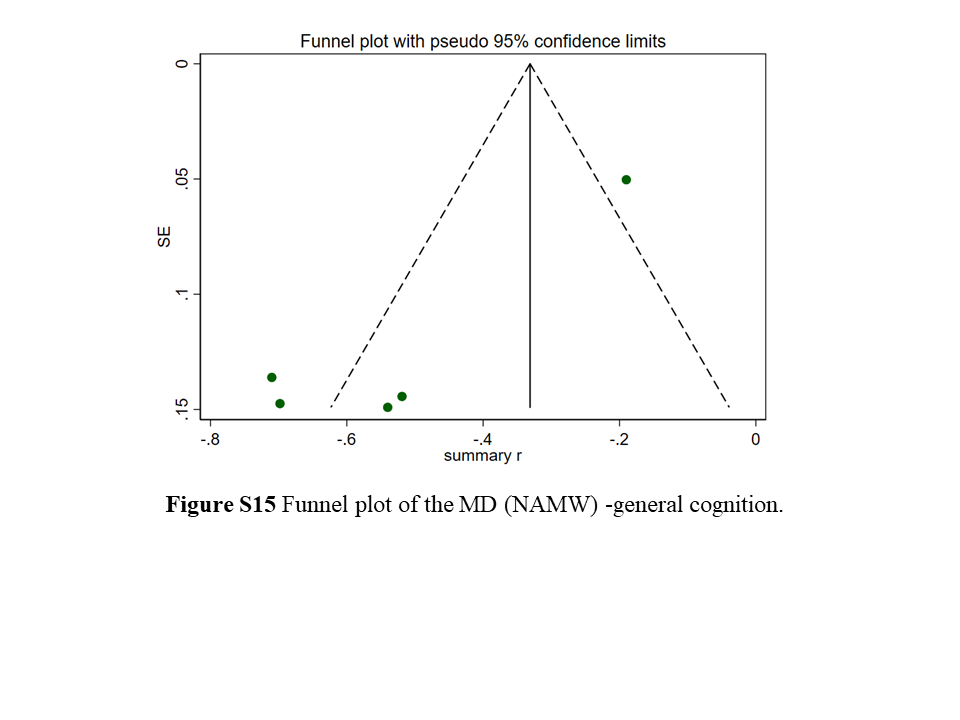

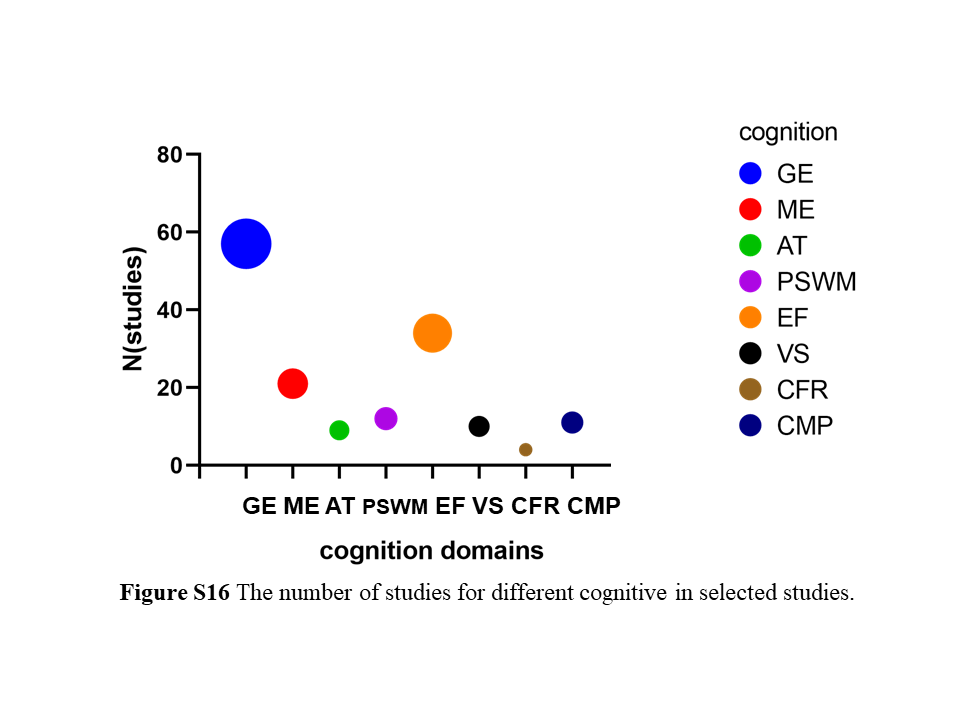

Supplement: Supplementary file 3 [file Data_Sheet_1.DOCX]
